# Octenidine-based hydrogel shows anti-inflammatory and protease-inhibitory capacities in wounded human skin

**DOI:** 10.1038/s41598-020-79378-9

**Published:** 2021-01-08

**Authors:** Saskia Seiser, Lukas Janker, Nina Zila, Michael Mildner, Ana Rakita, Johannes Matiasek, Andrea Bileck, Christopher Gerner, Verena Paulitschke, Adelheid Elbe-Bürger

**Affiliations:** 1 Department of Dermatology, Medical University of Vienna, Vienna, Austria; 2 Department of Analytical Chemistry, University of Vienna, Vienna, Austria; 3 Joint Metabolome Facility, University of Vienna and Medical University of Vienna, Vienna, Austria; 4 Department of Plastic, Aesthetic and Reconstructive Surgery, St. Josef Hospital, Vienna, Austria

**Keywords:** Infection, Interleukins

## Abstract

Octenidine dihydrochloride (OCT) is a widely used antiseptic molecule, promoting skin wound healing accompanied with improved scar quality after surgical procedures. However, the mechanisms by which OCT is contributing to tissue regeneration are not yet completely clear. In this study, we have used a superficial wound model by tape stripping of ex vivo human skin. Protein profiles of wounded skin biopsies treated with OCT-containing hydrogel and the released secretome were analyzed using liquid chromatography-mass spectrometry (LC–MS) and enzyme-linked immunosorbent assay (ELISA), respectively. Proteomics analysis of OCT-treated skin wounds revealed significant lower levels of key players in tissue remodeling as well as reepithelization after wounding such as pro-inflammatory cytokines (IL-8, IL-6) and matrix-metalloproteinases (MMP1, MMP2, MMP3, MMP9) when compared to controls. In addition, enzymatic activity of several released MMPs into culture supernatants was significantly lower in OCT-treated samples. Our data give insights on the mode of action based on which OCT positively influences wound healing and identified anti-inflammatory and protease-inhibitory activities of OCT.

## Introduction

Human skin has a variety of crucial functions such as serving as a barrier protecting the body from physical and chemical attack, invasion of pathogens and excessive water loss. As the primary immunological barrier to the environment, the skin is harboring several types of immune cells that participate in innate and adaptive immune responses^1,2^. Because skin is constantly exposed to potential injury, wound healing is a fundamental process for the survival of all higher organisms. Acute wound healing represents the physiological mode of wound closure and includes inflammation, blood clotting, cellular proliferation and extracellular matrix (ECM) remodeling^3,4^. In contrast, chronic wounds do not follow such a well-defined cascade, but are trapped in an inflammatory state^5^. Furthermore, such a continuously localized inflammation contributes to the pathogenesis of excessive wound healing, leading to the formation of keloids or hypertrophic scars. Pro-inflammatory cytokines have a major role in the first phases of wound healing. Interleukin (IL)-6 is enhancing the inflammatory response and induces vascular endothelial growth factor (VEGF) and IL-8 for tissue repair^6^. Further, IL-6 is favoring transition from acute to chronic inflammation as it exerts stimulatory effects on T and B cells^7^. During the remodeling phase a variety of proteolytic enzymes, such as matrix-metalloproteinases (MMPs), are active. They are capable of degrading the ECM, thus playing a major role in wound healing and tissue neomorphogenesis. MMP activity is mainly regulated via tissue inhibitors of metalloproteinases (TIMPs)^8^. In chronic wounds or wounds showing delayed wound healing frequently, an imbalance between TIMPs and MMPs has been observed^9,10^. Enhanced activity of MMPs might cause increased ECM degradation, alteration of the cytokine profile, and degradation of growth factors, culminating in delayed or absent wound closure^11^.

Octenidine dihydrochloride (OCT) is a widely used antiseptic molecule with antibacterial, antifungal and partially antiviral effects^12,13^. Due to the broad pH range in which OCT is active, it is perfectly suitable for wound care^14^. Additionally, OCT has the capacity to clear bacterial biofilms in patients with chronic wounds^15^. Recent studies revealed that OCT is promoting skin wound healing accompanied with improved scar quality after surgical procedures in patients^16^. Further, it has been demonstrated that OCT does neither morphologically alter skin cell architecture, nor enhance apoptosis when locally applied to superficially wounded ex vivo human skin. In addition, OCT prevents migration of epidermal Langerhans cells and inhibits the secretion of several cytokines (IL-8, IL-33, IL-10) in vitro, suggesting an anti-inflammatory capacity^17^.

Although animal models have the advantage of representing a whole organism, there are major anatomical and physiological differences, making a comparison to the human system often difficult^18^. Especially wound healing shows major differences between rodents and humans, underlining the necessity of alternative wound healing models. Ex vivo human skin models are of high relevance because they provide laboratory models perhaps closest to the in vivo environment in terms of biological complexity and fidelity to human physiology. Even though we are aware that the use of ex vivo human skin models has some limitations (e.g., dependency on biological material, limitations in standardization due to donor variability, lacking the influx of biochemicals [complement proteins from the circulation] and other immune cells [natural killer cells, neutrophils, T cells], adaptive responses cannot be tested, no innervation, limited culture periods), they allow comparative assessments on the whole skin compartment of one area and cell–cell interactions^19^. Further, ex vivo skin models are ethically advantageous, because they permit experiments in human tissue that would otherwise be impossible in living individuals. They are a simple, fast, and cost-effective tool for decreasing large-scale and expensive animal testing. Because there are increasing restrictions in Europe for using animals for testing compound properties and in line with the 3Rs (reduction, refinement, and replacement of animal models), ex vivo models represent an ideal preclinical platform. Therefore, we have employed a recently established ex vivo human wound model^17,20^ and a proteomic as well as ELISA-based approach to determine the protein profile of OCT-treated wounded skin, aiming to further unravel the largely unknown mechanism by which OCT might contribute to tissue regeneration and improved wound healing.

## Materials and methods

### Ethics statement

Abdominal skin from anonymous healthy adult female donors was obtained during plastic surgery procedures. The study was approved by the ethics committee of the Medical University of Vienna and conducted in accordance with the principles of the Declaration of Helsinki. A written informed consent was obtained from participants.

### Superficial ex vivo human skin wound model using tape-stripping

Freshly isolated skin (1–2 h (hours) after surgery) was disinfected with Kodan forte (Schülke & Mayr GmbH, Austria) and tape-stripped (TS), essentially as described previously^20^. Briefly, 50 different D102-squame standard self-adhesive discs (CuDerm, USA) were applied onto excised skin on the identical area by the same operator with constant pressure for 10 s (sec). Subsequently, the TS skin was cut with a Dermatome (0.6 mm; Acculan 3Ti; Aesculap, Inc. USA) and punch biopsies (∅ 8 mm; Kai Europe GmbH, Germany) were taken from TS and non-TS (NTS) skin. The efficient removal of the *stratum corneum* was verified by routine hematoxylin and eosin (H&E) staining of TS and NTS punch biopsies.

To define if the removal efficiency of the *stratum corneum* is comparable in all positions of a TS area, or shows differences, according to potential unequally applied pressure and donor variability, abdominal skin of three donors was TS and biopsies were taken from five defined regions on the TS area (Fig. S1). H&E staining demonstrated that TS is most efficient in the inner region (= 2) compared to outer regions (= 1, 3, 4, 5) (Fig. S1). Aiming to standardize further experiments as much as possible, only biopsies of the inner region were used in this study.

### Topical application of OCT and culture conditions

Before culture, OCT (octenilin wound gel, 0.05% OCT, Schülke & Mayr GmbH, Germany), or control gel (OCT-free hydrogel, Schülke & Mayr GmbH, Germany) were applied topically onto TS skin biopsies (50 microliter (µl)/biopsy). Untreated and treated TS skin biopsies were cultured in 12-well culture plates in the presence of Dulbecco´s modified eagle medium (DMEM; 500 µl/well; Gibco, Thermofisher, USA), supplemented with 10% fetal bovine serum (FBS; Gibco, Thermofisher) and 1% penicillin–streptomycin (Gibco, Thermofisher) for 48 h at 37 °C and 5% CO_2_ (Heracell 150i; Thermofisher, USA). On the second day, biopsies and culture supernatants were collected and stored at − 80 °C for liquid chromatography-mass spectrometry (LC–MS) and enzyme-linked immunosorbent assays (ELISA), respectively.

### Separation of skin compartments

In certain experiments, treated and untreated TS skin biopsies were washed in PBS upon 48 h of cultivation. To be able to separate the epidermis from the dermis, samples were enzymatically treated by incubation in 1.2 U/ml Dispase II (Roche Diagnostics GmbH) at 4 °C overnight. On the next day the epidermis was peeled from the dermis using forceps. Separated compartments were immediately frozen in liquid nitrogen without fixation and stored on − 80 °C for LC–MS analysis.

### H&E staining

Fixed (7.5% paraformaldehyde) TS and untreated skin samples were embedded in paraffin (Sanova Pharma GmbH, Austria), cut (5 μm) with a microtome (Microm HM 335 E; GMI, USA) and stained with H&E solution according to standardized protocols.

### Sample preparation and LC–MS shotgun analysis of whole TS skin biopsies

Cultured TS biopsies (female, abdomen, age range: 38–45 years, n = 5) treated with OCT, control gel and left untreated, were homogenized in 100 μl sample buffer (7.5 molar (M) urea, 1.5 M thiourea, 0.1 M dithiothreitol, 4% 3-[(3-cholamidopropyl)dimethylammonio]-1-propanesulfonate, 0.05% sodium dodecyl sulfate) using ultrasound. Protein concentrations were premeasured using a Bradford assay. For each sample 20 µg protein was used for the in-solution digest performed according to a variation^21^ of the FASP protocol^22^. Dried samples were reconstituted as previously described^23,24^ in 5 µl 30% formic acid (FA) containing 4 synthetic standard peptides and diluted with 40 µl mobile phase A (97.9% H_2_O, 2% ACN, 0.1% FA). Of this solution, 5 µl were injected into a Dionex Ultimate 3000 nano high performance liquid chromatography (HPLC)-system (Thermo Fisher Scientific). Peptides were concentrated on a pre-column (2 cm × 75 µm C18 Pepmap100; Thermo Fisher Scientific) at a flow rate of 10 µl/min, using mobile phase A. Subsequently, they were separated by elution from the pre-column to an analytical column (50 cm × 75 µm Pepmap100; Thermo Fisher Scientific) applying a flow rate of 300 nl/min and using a gradient of 8% to 40% mobile phase B (80% ACN, 20% H_2_O, 0.1% FA), over 190 min for sample analysis. The MS analysis was performed on a QExactive classic orbitrap mass spectrometer, equipped with a nanospray ion source (Thermo Fisher Scientific), coupled to the nano HPLC system. For detection, MS scans were performed in the range from m/z 400–1400 at a resolution of 70.000 (at m/z = 200). MS/MS scans were performed choosing a top 8 method. HCD fragmentation was applied at 30% normalized collision energy and analysis in the orbitrap at a resolution of 17.500 (at m/z = 200).

### LC–MS data analysis and interpretation of whole TS skin biopsies

Analysis and interpretation of LC–MS data was performed essentially as described^21^ using the open source software MaxQuant (version 1.6.3.4)^25,26^ including the Andromeda search engine and the Perseus statistical analysis package^25,26^. Protein inference was achieved aligning against homo sapiens in the SwissProt Database setting the parameters (mass tolerance, missed cleavages, fixed/variable modifications, minimum of peptide identifications) as defined^21^. Label-free quantification (LFQ) revealed values for each individual protein, which were conducted for quantitative assessment of protein regulation using the Perseus statistical analysis package^27^. Proteins differentially expressed between the three treatments were evaluated via Perseus with an unpaired t-test using LFQ values of proteins for each group. Proteins with a *p*-value lower than 0.05 and a difference lower than − 1 or higher than + 1, corresponding to a log_2_ fold change of at least 2, were considered as significant hits and were further analysed. Protein function was determined using the Uniprot database^28^ as well as DAVID bioinformatics resources for gene ontology annotation^29,30^ .

### Sample preparation and LC–MS analysis of epidermal and dermal compartments

Cultured TS biopsies (female, abdomen, age range: 36–45 years, n = 3) treated with OCT, control gel and left untreated, were enzymatically separated into dermis and epidermis and the different compartments were independently homogenized in 100 μl lysis buffer (8 M urea, 50 mM TEAB, 5% SDS) using ultrasound. For enzymatic digestion, a protocol using the S-trap technology was employed^31^. In short, reduction and alkylation of 20 µg solubilized protein amount was achieved with 64 mM DTT and 48 mM IAA, respectively. After addition of trapping buffer (90% vol/vol Methanol, 0.1 M Triethylammonium bicarbonate), samples were loaded onto S-trap cartridges, thoroughly washed and subsequently digested using Trypsin/Lys-C Mix at 47 °C for one hour. Peptides were eluted, dried and stored at − 20 °C until LC–MS analyses. Dried samples were reconstituted in 5 µl of 30% FA containing 4 synthetic standard peptides and diluted with 40 µl of loading solvent (97.9% H_2_O, 2% ACN, 0.05% trifluoroacetic acid). Of this solution, 5 µl were injected into the Dionex Ultimate 3000 nano HPLC-system (Thermo Fisher Scientific). Peptides were concentrated on a pre-column (2 cm × 75 µm C18 Pepmap100; Thermo Fisher Scientific) at a flow rate of 10 µl/min, using mobile phase A (97.9% H_2_O, 2% ACN, 0.1% FA). Subsequently, they were separated by elution from the pre-column to an analytical column (50 cm × 75 µm Pepmap100; Thermo Fisher Scientific), applying a flow rate of 400 nl/min and using a gradient of 8% to 40% mobile phase B (80% ACN, 20% H_2_O, 0.1% FA) over 95 min, resulting in a total LC run time of 135 min including washing and equilibration steps. Mass spectrometric analyses were accomplished using the timsTOF Pro mass spectrometer (Bruker) equipped with a captive spray ion source run at 1600 V. Furthermore, the timsTOF Pro mass spectrometer was operated in the Parallel Accumulation-Serial Fragmentation (PASEF) mode. Trapped ion mobility separation was achieved by applying a 1/k0 scan range from 0.60 to 1.60 V^.^s/cm^2^ resulting in a ramp time of 166 ms. All experiments were performed with 10 PASEF MS/MS scans per cycle leading to a total cycle time of 1.88 s. MS and MS/MS spectra were recorded using a scan range (m/z) from 100 to 1700. Further, the collision energy was ramped as a function of increasing ion mobility from 20 to 52 eV and the quadrupole isolation width was set to 2 Th for m/z < 700 and 3 Th for m/z > 700.

### LC–MS data analysis and interpretation of epidermal and dermal compartments

For ion mobility mass spectrometry (IMMS) data, the software pipeline FragPipe (version 13.0) employing the search algorithm MSFragger (version 3.0)^32^ and Philosopher (version 3.2.7) for peptide and protein identification statistics in combination with IonQuant (version 1.3.0) was used. Mass tolerance was set to 50 ppm and 20 ppm for MS1 and MS2, respectively. One missed cleavage was allowed. Fixed/variable modifications and peptide identification settings were set as described above. For quantification of proteins, a MaxLFQ approach with IonQuant was chosen. “Match-between-runs” (MBR) parameters were set to a retention time window of 1 min and an ion mobility window of 0.05 1/K_0_. A MBR false discovery rate cut-off value of 0.01 on ion, peptide and protein level was applied. Statistical analysis of obtained results was performed with Perseus statistical analysis package (version 1.6.14.0), as described above. Proteins with a *p*-value lower than 0.01 and a difference lower than − 1 or higher than + 1, corresponding to a log_2_ fold change of at least 2, were considered as significant hits and were further analysed. Protein function was determined using the Uniprot database as well as DAVID bioinformatics resources for gene ontology annotation.

### Data availability

The MS proteomics data have been deposited to the ProteomeXchange Consortium via the PRIDE^33^ partner repository with the dataset identifier PXD007592 and https://doi.org/10.6019/pxd007592.

### ELISA

Culture plates (Nunc Immuno 96 well flat-bottom culture plate; Merck KGaA) were coated with appropriate capture human antibodies such as IL-8 (Thermo Fisher Scientific, #M108), IL-6, MMPs or TIMP-1 (R&D Systems) overnight at 4 °C or room temperature. On the next day, plates were washed with washing buffer (0.05% Tween in PBS) and blocked with blocking buffer (4% BSA, 0.5% Tween in PBS) or reagent diluent (1% BSA in PBS). Another washing step was performed, and standards and samples were pipetted on the plates in an appropriate dilution and incubated for 2 h at room temperature. After that, plates were aspirated and the respective detection antibody was applied (Thermo Fisher Scientific, #M802B; R&D Systems) and incubated for another 2 h at room temperature. Next, plates were washed again and streptavidin-horse-radish peroxidase (Thermo Fisher Scientific #34028, R&D Systems) was applied. After an incubation time of 20 to 30 min, another washing step was performed and 3,3′,5,5′-tetramethylbenzidine substrate reagent A + B (BD Biosciences, USA) was applied and incubated for 20 min. Subsequently, a stop solution (2N H_2_SO_4_; 50 µl/well) was added and fluorescence was measured immediately at 450 nm with a photometer (Multiskan FC Microplate Photometer; Thermofisher, USA).

### Enzyme activity assay

The activity of MMPs (MMP1/2/3/7/8/9/10/12/13) in culture supernatants was measured using the Amplite Universal Fluorimetric MMP Activity Assay Kit (AAT Bioquest, USA). Supernatants were activated with 4-aminophenylmercuric acetate (2 mM) overnight. Activated supernatants and controls were pipetted in duplicates into a flat-bottom culture plate (100 µl/well; Corning 96 Well White Polystyrene Microplate; Merck KGaA) and MMP Green substrate (50 µl/well; AAT Bioquest) was added. Fluorescence intensity was monitored as kinetic measurement with a plate reader (BMG FLUOstar OPTIMA Microplate Reader; BMG Labtech Inc. USA) at Ex/Em = 490/525 nm.

### Statistical analysis

ELISA data as well as results from the enzyme activity assay were analyzed using GraphPad Prism 5. An unpaired t-test was used for comparing means, respectively. The results were considered significant with *p*-values smaller than 0.05. Detailed information about statistical evaluation of LC–MS analysis and interpretation can be found in the respective method sections.

## Results

### OCT-treated wounded human skin shows a distinct proteomic profile compared to controls

**Figure 1:**
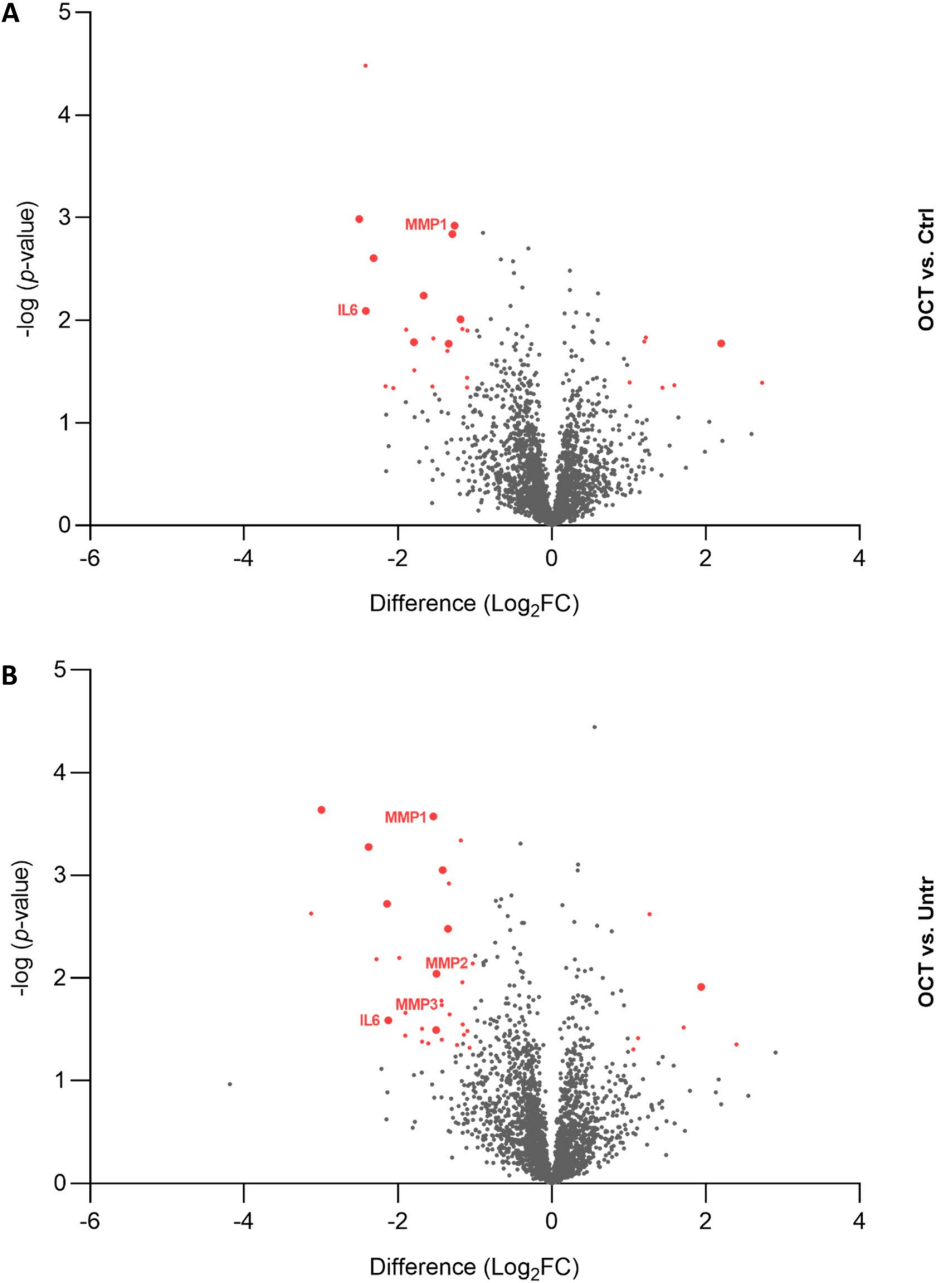
Proteins are differentially regulated among wounded OCT-treated skin and controls. Abdominal skin biopsies of donors (age range: 38–45 years, n = 5) were TS, treated with OCT, control gel or left untreated, cultivated for 48 h and subsequently prepared for LC–MS analysis. Volcano plots show differences in LFQ values (fold change, logarithmic scale to the base of two) on the x-axis including their corresponding *p*-values (logarithmic scale) on the y-axis. Proteins marked in red are significantly differentially regulated (Log_2_FC < -1 = downregulated; Log_2_FC > 1 = upregulated) in OCT treated samples compared to **(A)** control gel-treated samples or **(B)** untreated samples. Larger sized dots indicate proteins that are differentially regulated in OCT treated samples compared to both, control gel-treated samples and untreated samples. Registered proteins were further analysed. Data presented in the plots was generated performing a two-sided t-test (*p* < 0.05) using Perseus and vulcano plots were generated using Graphpad Prism. Extended information on the proteins can be found in Tables S1 and S2.

To uncover the protein profile of human skin cells upon wounding and OCT-treatment, a proteomic approach was chosen. Abdominal TS skin biopsies of five donors were treated with OCT, control gel or left untreated, cultivated for 48 h and subjected to LC–MS. Statistical analysis using the software packages MaxQuant and Perseus revealed a total of 2622 proteins (FDR < 0.01). Twenty-six proteins were found to be differentially regulated (FDR < 0.05) between OCT- and control gel-treated samples (Fig. 1A, Table S1) and thirty-seven proteins were differentially regulated between OCT-treated and untreated samples (Fig. 1B, Table S2). Ten proteins were differentially regulated between both, OCT-treated and untreated samples and between OCT-treated and control-treated samples (Fig. 1A,B). Additionally, sixteen proteins were differentially regulated between the untreated and control gel-treated group, showing that already application of hydrogel on the wound has an effect on the protein profile of skin cells (Table S3). Among those proteins, three proteins were also differentially regulated between OCT-treated and untreated samples and two proteins between OCT-treated and control-gel treated samples (Fig. S2). Of note, none of the ten proteins which were differentially regulated between both, OCT-treated and control gel-treated and OCT-treated and untreated samples were among the sixteen proteins differentially regulated between the untreated and control-gel-treated group, concluding that OCT indeed has a regulatory effect on respective proteins. Among significant hits, several proteins are involved in the immune response as well as wound healing and tissue regeneration (Tables S1 and S2).

### OCT regulates biological processes relevant for wound healing and immune response

**Figure 2:**
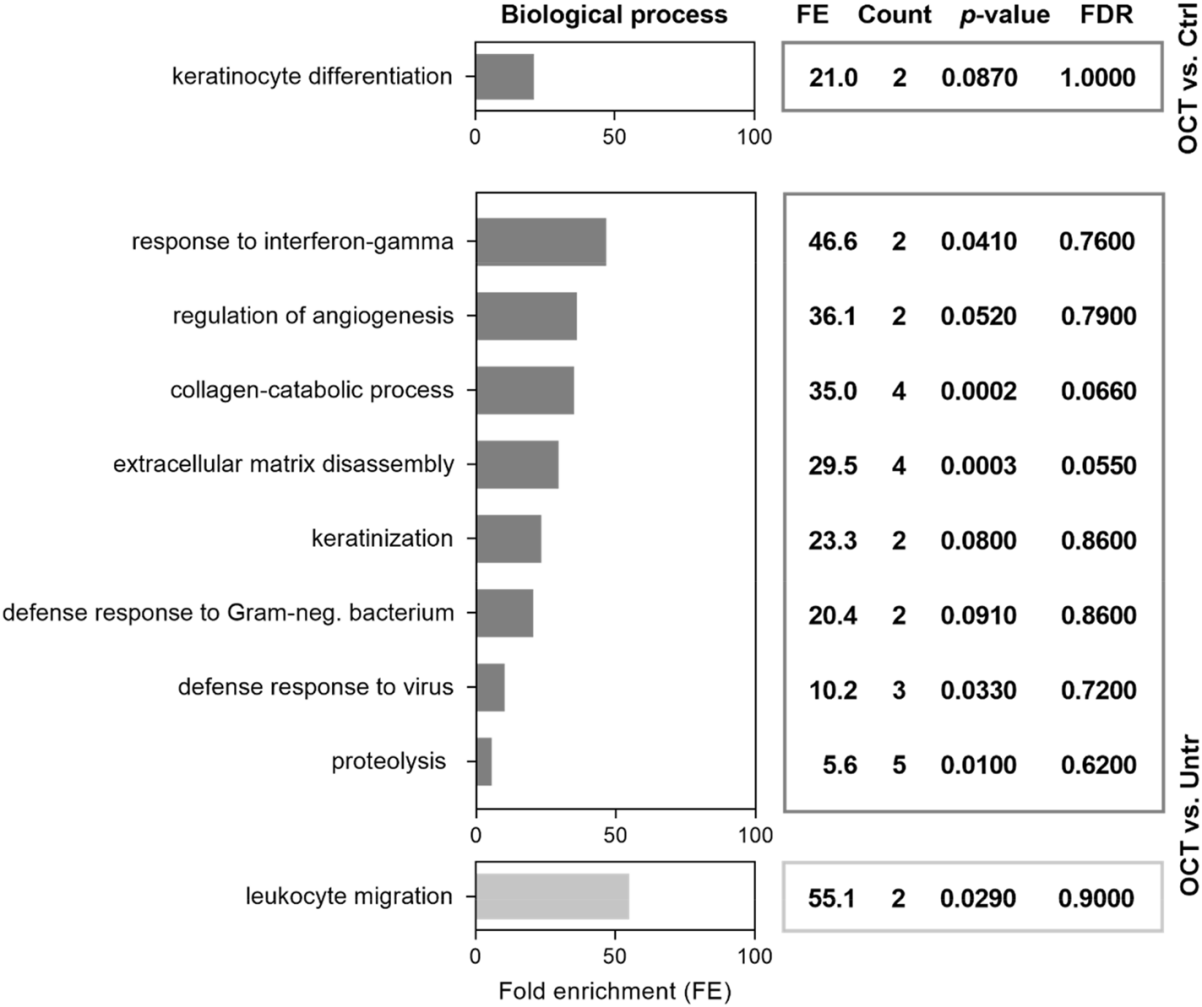
OCT regulates proteins involved in wound healing and immune response. Full skin biopsies of donors (age range: 38–45 years, n = 5) were TS, treated with OCT, control gel or left untreated, cultivated for 48 h and subsequently prepared for LC–MS analysis. Classification by the GO term biological process shows pathways and larger processes made up of the activities of multiple proteins. Fold enrichment values for individual GO terms, count (proteins involved in the term), *p*-value and FDR (false discovery rate, calculated using the Benjamini–Hochberg procedure), listed next to the graph, were calculated using DAVID bioinformatics resources. Biological processes downregulated in OCT-treated TS samples are shown in dark grey, processes upregulated in OCT-treated TS samples are shown in light grey.

Gene Ontology (GO) enrichment analysis of proteins differentially regulated in OCT-treated wounded skin was performed using DAVID Bioinformatics Resources^29,30^. We found that proteins downregulated in OCT-treated TS skin were associated with the biological functions important for wound healing (Fig. 2). Angiogenesis, collagen catabolic process, ECM disassembly as well as proteolysis were downregulated in OCT-treated TS skin compared to untreated wounded skin (Fig. 2). Proteins involved in those functions are, among others, IL-6 and members of the MMP family (MMP1, 2, 3). As anticipated, also proteins related to functions involved in inflammatory immune response, such as particular defense mechanisms were downregulated in OCT-treated wounded skin samples (Fig. 2). According to GO, IL-6 is an important representative of those functions. Together, these data suggest, that OCT reduces the inflammatory immune response and excessive collagen and ECM deposition in wounded skin. Leukocyte migration was upregulated in TS skin biopsies upon OCT treatment (Fig. 2), showing that OCT not only has suppressing capacities. Functional associations between proteins downregulated in OCT treated wounded skin samples were determined using the STRING software^34^. The analysis revealed strong functional interaction between members of the MMP family (MMP1, 2, 3) and IL-6 (Fig. S3), conforming and extending studies demonstrating that IL-6 regulates tissue MMPs^35,36^.

### OCT significantly reduces secretion of pro-inflammatory cytokines in wounded human skin

**Figure 3:**
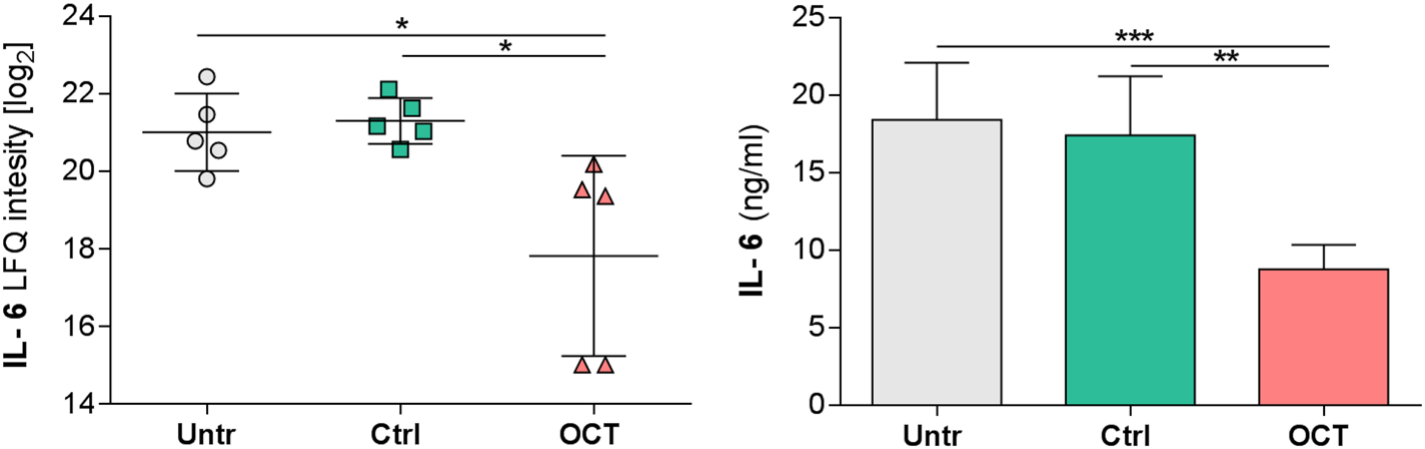
OCT significantly inhibits IL-6 secretion in wounded human skin. IL-6 levels of TS human skin biopsies treated with OCT were evaluated by LC–MS and were significantly lower compared with TS biopsies which were untreated or treated with the control gel (left panel). Label-free quantification (LFQ) intensities in a logarithmic scale to the basis 2 are indicated. LFQ intensities for proteins not detected in a replicate were replaced by 15. IL-6 concentration in supernatants was tested in duplicates with an ELISA (right panel). Data in both graphs is presented as a mean ± SD (n = 5). An unpaired t-test was performed with GraphPad Prism. *p ≤ 0.05, **p ≤ 0.01, ***p ≤ 0.001.

Differentially regulated protein candidates in OCT-treated TS skin samples and controls were further quantitatively assessed by ELISA. In addition to IL-8 (Fig. S4)^17^*,* IL-6 is another pro-inflammatory cytokine known to play a key role in acute inflammation and is pivotal for the timely resolution of cutaneous wound healing^37^. Dysregulation of IL-6 signaling can lead to either fibrosis or a failure to heal. Our LC–MS data are in line with this concept as we found significantly diminished IL-6 in OCT-treated compared to untreated or control gel-treated TS skin biopsies (Fig. 3). Analysis of culture supernatants of the same donors confirmed these data showing significantly reduced IL-6 concentrations in OCT-treated wounded skin samples compared to controls, supporting and extending our previous conclusion that OCT has anti-inflammatory capacity (Fig. 3).

### OCT downregulates release of proteases in wounded human skin

**Figure 4:**
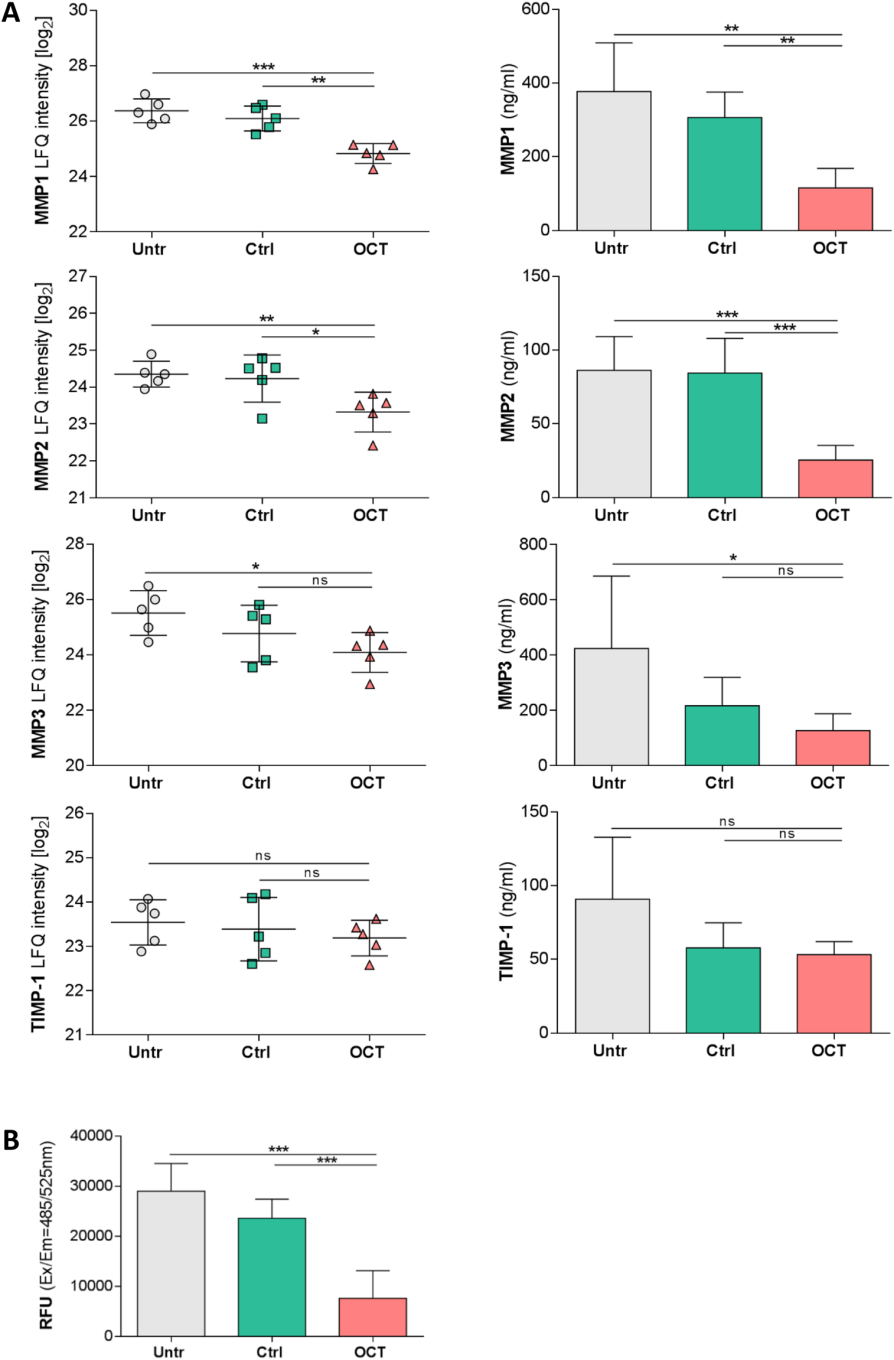
OCT significantly reduces secretion and activity of proteases in wounded human skin. **(A)** Indicated MMP levels, analysed by LC–MS, were significantly lower in human TS skin biopsies treated with OCT compared to biopsies which were untreated or treated with a control gel, with exception of TIMP-1, which is not affected upon OCT treatment (left panel). Label-free quantification (LFQ) intensities in a logarithmic scale to the basis 2 are indicated. LFQ intensities for proteins not detected in a replicate were replaced by 15. Proteases and a typical protease inhibitor in culture supernatants were tested in duplicates with an ELISA (right panel). **(B)** Enzymatic activity of MMPs was significantly lower in culture supernatants of OCT-treated TS human skin biopsies compared to controls (untreated, control gel-treated). Relative fluorescence units (RFU) are indicated. All data is presented as mean ± SD (n = 5). An unpaired t-test was performed with GraphPad Prism. *p ≤ 0.05, **p ≤ 0.01, ***p ≤ 0.001, ns = not significant.

MMPs and their inhibitors play a crucial role in all stages of wound healing by regulating ECM degradation and deposition which allows tissue remodelling as well as cell migration^38^. We found that MMP1 and MMP2 protein levels were significantly lower in the tissue and in the secretome of TS skin biopsies treated with OCT in comparison to controls (Fig. 4A). MMP3 expression and secretion levels were significantly lower only in OCT-treated wounded skin samples when compared to untreated samples (Fig. 4A). Of note, OCT treatment of TS human skin did not influence TIMP-1 expression and secretion (Fig. 4A). Importantly, supernatants of TS biopsies of the same five donors treated with OCT showed significantly lower total MMP activity than supernatants of control groups (Fig. 4B).

### OCT is primarily active in the dermal compartment

**Figure 5:**
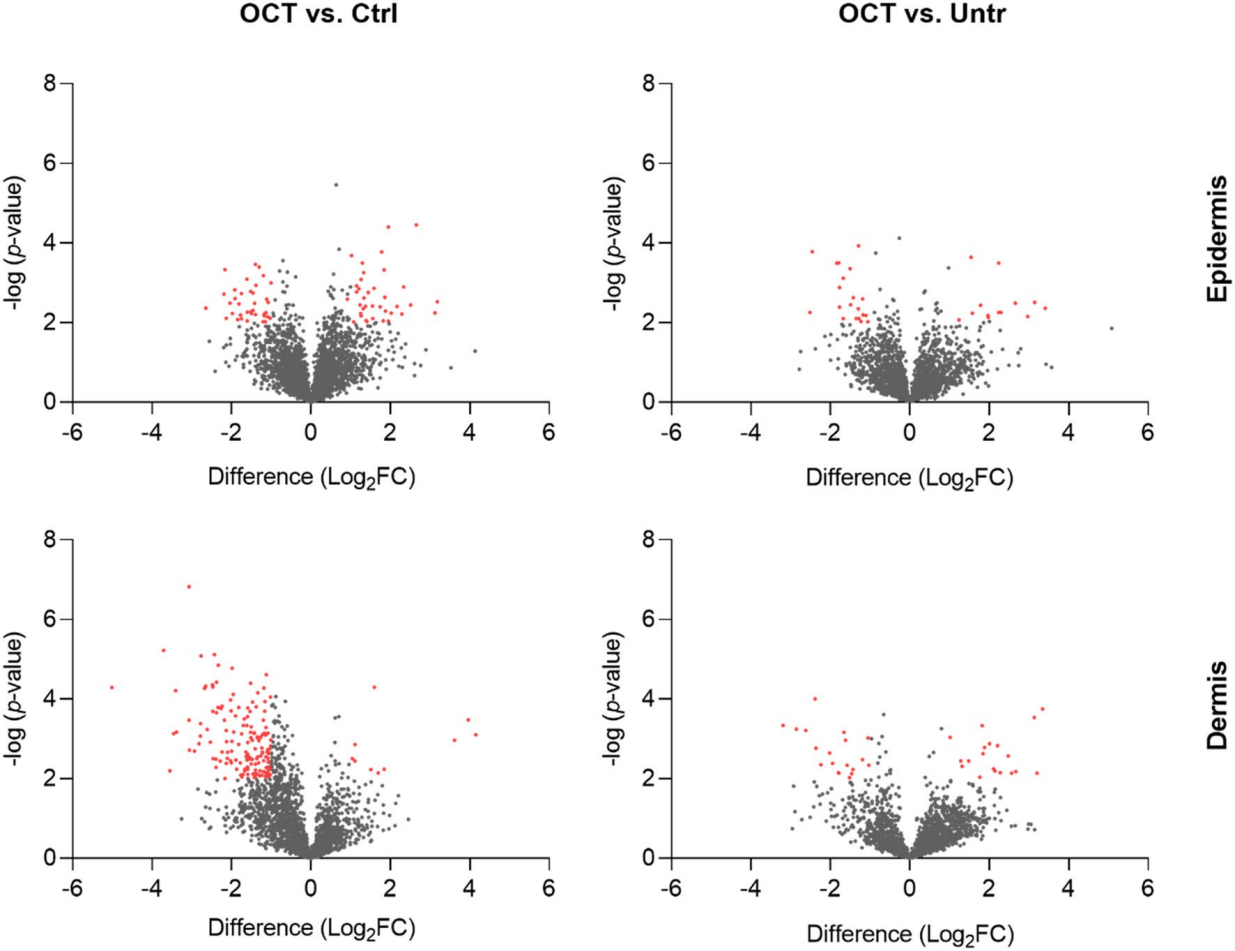
More proteins are significantly differentially regulated upon OCT treatment in the dermis than in the epidermis. Full skin biopsies of healthy female donors (age range: 36–44 years, n = 3) were TS, treated with OCT, control gel or left untreated and cultivated for 48 h. Subsequently, epidermis was detached from dermis and the two compartments were separately prepared for LC–MS analysis. Volcano plots show differences in LFQ values (fold change, logarithmic scale to the base of two) on the x-axis including their corresponding *p*-values (logarithmic scale) on the y-axis. Proteins marked in red are significantly differentially regulated (Log_2_FC < -1 = downregulated; Log_2_FC > 1 = upregulated) in OCT treated TS samples compared to control gel-treated TS samples or untreated TS samples. Data presented in the plots was generated performing a two-sided t-test (*p* < 0.05) using Perseus and Volcano plots were generated with Graphpad Prism using exclusively proteins with a *p*-value < 0.01.

**Figure 6:**
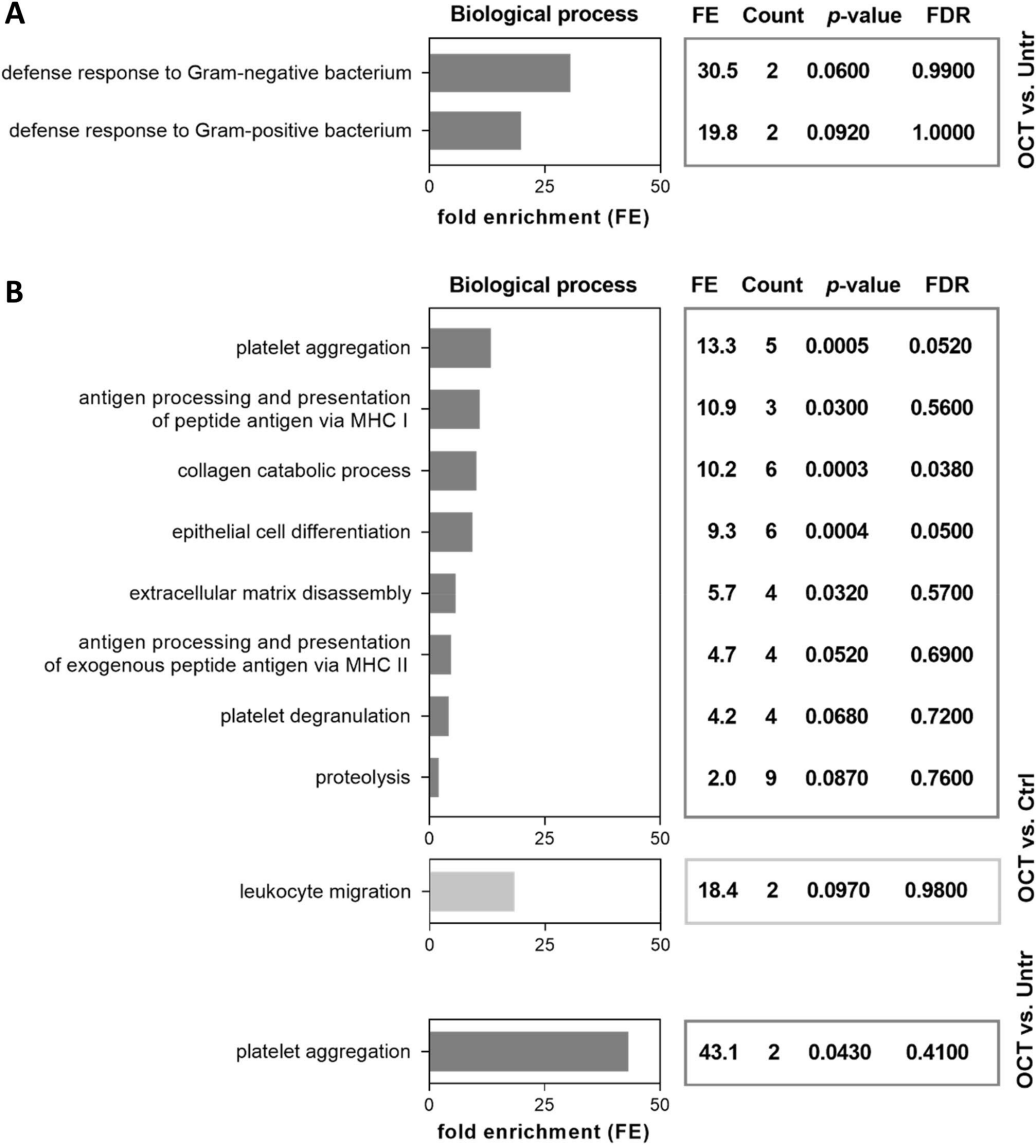
OCT regulates proteins involved in immune response in the epidermal compartment and proteins involved in wound healing and immune response in the dermal compartment. Full skin biopsies of donors (age range: 36–44 years, n = 3) were TS, treated with OCT, control gel or left untreated and cultivated for 48 h. Subsequently, epidermis **(A)** was detached from dermis **(B)** and the two compartments were separately prepared for LC–MS analysis. Classification by the GO term biological process shows pathways and larger processes made up of the activities of multiple proteins. Fold enrichment values for individual GO terms, count (proteins involved in the term), *p*-value and FDR (false discovery rate, calculated using the Benjamini–Hochberg procedure), listed next to the graph, were calculated using DAVID bioinformatics resources. Biological processes downregulated in OCT-treated TS samples are shown in dark grey, processes upregulated in OCT-treated TS samples are shown in light grey.

As MMPs and inflammatory cytokines are produced and secreted by many cell types^24^, we next investigated whether the protease-inhibitory and anti-inflammatory action is occurring preferentially in the epidermis or dermis. To address this, TS skin biopsies of a different set of three healthy female donors, as before, were treated with OCT, control gel or left untreated and cultivated for 48 h. Physically separated epidermal and dermal compartments were then analysed with complementary instrument technology employing ion mobility gas phase separation. Statistical analysis revealed that more proteins were differentially regulated in OCT-treated TS skin samples compared to control gel-treated samples than in comparison to untreated samples. Within both groups, more proteins were differentially regulated in the dermis (OCT/Ctrl: 173; OCT/Untr: 38) than in the epidermis (OCT/Ctrl: 73; OCT/Untr: 34) (Fig. 5). Of note, the disparity between the two compartments is clearly larger when OCT-treated TS skin was compared with control gel-treated TS skin (Fig. 5). Only few proteins were differentially regulated between control gel-treated and untreated samples, in the epidermis (2) as well as dermis (6) (Table S8). Protein function was determined using the Uniprot database as well as DAVID Bioinformatics Resources. Again, among significant hits, several proteins are involved in immune response as well as wound healing (Fig. 6). In the epidermis (Fig. 6A) we found a downregulation of proteins involved in defense mechanisms in OCT-treated samples compared to untreated samples, being in conformity with the results of whole TS skin biopsy analysis (Fig. 2). In the dermis (Fig. 6B) several functions associated with wound healing were downregulated in OCT-treated samples compared to control gel-treated samples. Among them were also processes found in GO annotation results of whole TS skin biopsies (collagen catabolic process, ECM disassembly, proteolysis) (Fig. 2), which are predominantly related to members of the MMP family. Functions of all proteins significantly up- or downregulated upon OCT treatment, in both epidermis and dermis were determined using the Uniprot database (Tables S4, S5, S6, S7). Similar to data in Tables S1 and S2, significant differences were identified for MMP1 and MMP2 (Table S6) between OCT-treated and control gel-treated TS dermal samples. In addition, MMP9 was shown to be significantly downregulated in OCT-treated TS samples compared to the control (Table S6).

## Discussion

In clinical practice, OCT has been shown to promote healing of chronic wounds^39–41^ and to reduce the formation of hypertrophic scars in patients after abdominoplasty^16^. However, the mechanism by which OCT contributes to tissue regeneration and improved wound healing is not yet investigated in more detail. We used a mild, ex vivo human wound model to address this and applied standardization, according to sex and donor age range within one experimental set up, employing skin always from a distinct body location, defining the best TS area within a skin biopsy, and evaluating optimal skin thickness for subsequent analysis.

A detailed characterization of the protein profile of wounded and OCT-treated TS skin biopsies and controls was achieved using LC–MS. Out of 2622 identified proteins, 26 and 37 were differentially regulated between OCT- and control gel-treated samples as well as OCT-treated and untreated samples, respectively. GO annotations revealed several biological processes involved in wound healing among proteins found to be downregulated in OCT-treated skin and included ECM disassembly, collagen-catabolic process, proteolysis as well as angiogenesis. According to DAVID bioinformatics resources, proteins associated with above mentioned processes were among others, IL-6 and MMP family members. IL-6 is playing a key role in the acute phase reaction in response to immunological stress. Fetal skin, in contrast to adult skin, produces rather low levels of pro-inflammatory cytokines (IL-6, IL-8), potentially related to the immature immune status but attributing to its high regenerative capacity. Further, it has been reported that (1) exogenous IL-6 leads to scar formation of human fetal skin grafts in SCID mice^42^, (2) IL-8 levels are significantly increased in slowly healing human burn wounds, (3) IL-8 decreases keratinocyte differentiation in vitro^43^, and (4) fibroblasts from keloid scars show increased IL-8 levels compared with normal human fibroblasts^44^. This supports a possible contribution of IL-6 and IL-8 to pathological scar formation. Our data in this study showing significantly lower IL-6 levels in OCT-treated biopsies compared to controls extend our previously reported finding about its anti-inflammatory capacity^17^. The inhibition of IL-6 and IL-8 in OCT-treated TS skin biopsies is one possible explanation for the potency of OCT to enhance wound healing and prevent hypertrophic scar formation in vivo. MMPs are other candidates as they are capable of dissolving the basal lamina and lysis of surrounding tissue, thus facilitating proliferation and cell migration throughout acute wound healing. Dysregulation of MMP levels might lead to increased ECM degradation, alteration of the cytokine profile, and degradation of growth factors, culminating in delayed wound closure^11^. In line with this assumption is the observation that mRNA expression of MMP2 is highly increased in different types of human scar tissue when compared with normal tissue derived from the same patient^9^. Our data revealed significantly lower MMP1 and MMP2 levels in cultured wounded human skin treated with OCT in comparison to controls. In conformity with this observation, we noted a significant reduction of MMP3 for OCT-treated TS biopsies when compared to untreated TS biopsies, but not with TS biopsies treated with the control gel, suggesting that application of the hydrogel on the wound already leads to a decrease of MMP3 secretion. Our further finding that MMP activity was significantly reduced in OCT-treated samples compared to controls demonstrated that OCT has indeed a regulatory effect regarding protease inhibitors. Skin fibroblasts have been demonstrated to secrete IL-6 and IL-8 in addition to several chemokines, MMPs and protease inhibitors^45^. The dermis is rich in fibroblasts suggesting that these cells may be the most relevant cell type responding to OCT treatment. The fact that OCT inhibits the release of tissue MMPs might be one further explanation of the pathological scar preventing and wound healing promoting effects of OCT in vivo. TIMP-1 is one of the major MMP inhibitors, usually significantly downregulated in chronic or slowly healing wounds^10^. As OCT did not regulate TIMP-1, as revealed by LC–MS and ELISA analysis, the exact mechanism of MMP inhibition by OCT in our model is not yet clear and remains to be further investigated.

To address which skin compartment is influenced by the topical application of OCT onto TS human skin, the epidermis and dermis were not only analyzed separately but, in addition, with a more sensitive method. Less proteins were found to be differentially regulated in OCT-treated TS epidermal samples, while we found OCT-mediated protein regulation largely in the dermis. In line with our results is the finding that OCT significantly reduces MMP1 and MMP2 compared to control gel-treated dermal TS biopsies. The discovery that MMP9 is significantly reduced in OCT-treated TS dermal samples further supports our previous finding that OCT has protease-inhibitory capacities.

Today, moist wound healing is widely accepted as the state-of-the-art in professional wound care. The high water content of hydrogels allows them to benefit cutaneous healing essentially by supporting a moist wound environment and promoting autolytic debridement^46^. We have shown in all experiments herein that the combination of a hydrogel with the antiseptic OCT provides additional immunomodulatory features compared to the application of the very same hydrogel without any bioactive agent. The observed local anti-inflammatory environment in TS skin samples treated with an OCT containing hydrogel can be perfectly linked to the presence of the antiseptic itself in the gel and are not the result of pure moist wound healing alone.

**Figure 7:**
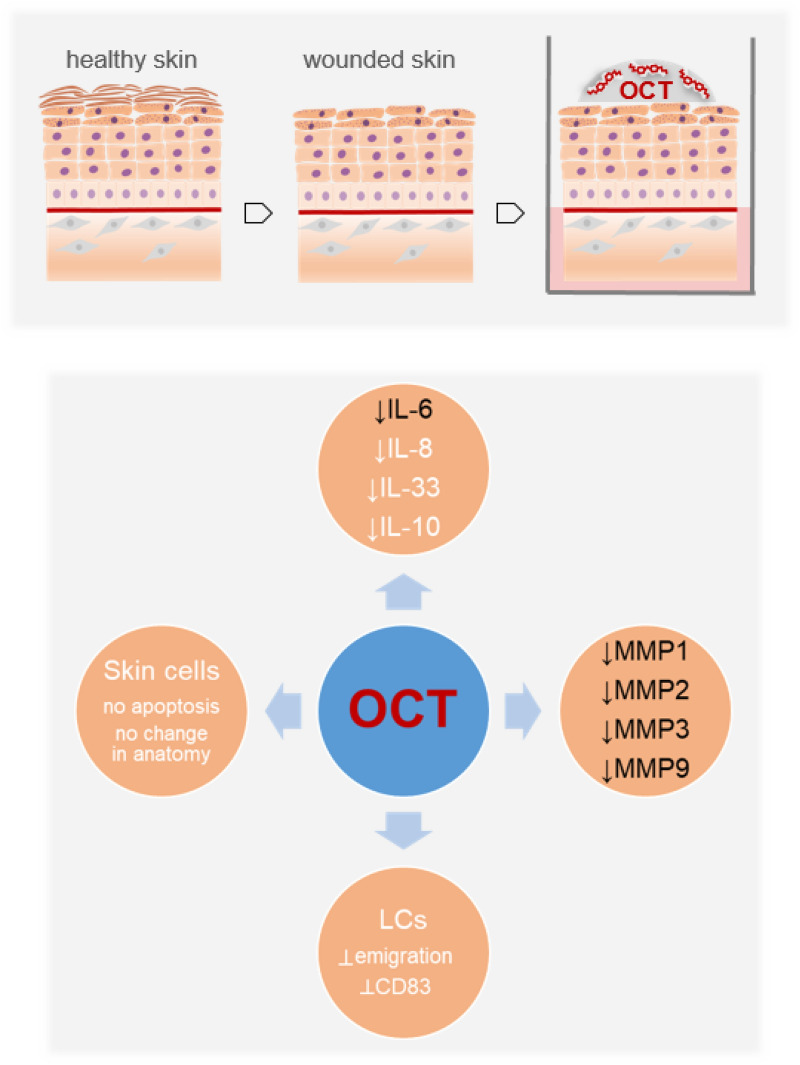
OCT reduces cytokines as well as protease activities in wounded human skin (this study, black font), is not apoptotic for skin cells, does not change the skin architecture and prevents the upregulation of CD83 on Langerhans cells as well as their emigration (white font)^17^.

To conclude, our data provide further new insights into the mode of action by which OCT improves wound healing in clinical settings, as we reported anti-inflammatory and protease-inhibitory activities of OCT in wounded human skin (Fig. 7) and identified the dermal proteome as its main target.

## Supplementary Information

Supplementary legends.

Supplementary figures and tables.

## Data Availability

Data, in anonymous format (according to data protection policy in the ethics agreement) is available on reasonable request.
