# Supplementary material for: Octenidine-based hydrogel shows anti-inflammatory and protease-inhibitory capacities in wounded human skin
Source: Sci Rep. 2021 Jan 8;11:32. doi: 10.1038/s41598-020-79378-9 (PMC7794247; doi:10.1038/s41598-020-79378-9)
Supplement: Supplementary file 1 — Supplementary legends. [file 41598_2020_79378_MOESM1_ESM.docx]

**SUPPLEMENTARY FIGURE LEGENDS**

**Figure S1. Complete removal of the *stratum corneum* in the inner region of the TS area.** Freshly isolated abdominal skin (female donors; age range: 33-38 years; n=3) was TS 50 times on the same area. Representative H&E staining (age 37 years) revealed that the most efficient removal of the *stratum corneum* is achieved in the inner region (=2) of a TS area. Biopsy positions correspond to the numbers indicated in the circle displayed in the upper left corner. NTS = non-tape stripped (control), TS-tape stripped. Scale bar = 50 µm.

**Figure S2. Intersections of differentially regulated proteins upon different treatments (OCT, control gel, untreated).** The Venn diagram shows that ten proteins are differentially regulated between OCT-treated and control gel-treated (OCT/Ctrl) as well as between OCT-treated and untreated samples (OCT/Untr). Among proteins differentially regulated between OCT-treated and untreated samples (OCT/Untr), three are differentially regulated between control-gel treated and untreated samples (Ctrl/Untr). Among proteins differentially regulated between OCT-treated and control gel-treated samples (OCT/Ctrl), two proteins are differentially regulated between control gel-treated and untreated samples (Ctrl/Untr). There is no intersection of differentially regulated proteins between all three groups (OCT/Ctrl, OCT/Untr, Ctrl/Untr). Diagrams were created using Venny 2.1.0 (https://bioinfogp.cnb.csic.es/tools/venny/).

**Figure S3.** **STRING network analysis shows strong functional interactions between MMPs and IL-6**. Shown are functional connections between a set of 37 proteins revealed to be downregulated in OCT treated human TS skin biopsies compared to untreated biopsies, generated with the STRING website. The minimum required interaction score was set to 0.700.

**Figure S4. OCT significantly inhibits IL-8 secretion in wounded human skin.** Shown are IL-8 concentrations in culture supernatants of 48 h cultured human TS skin biopsies (female donors; abdominal skin; age range: 27-52 years) that were topically treated with OCT (0.05% OCT in gel form), control gel (OCT-free hydrogel), and left untreated. IL-8 levels were tested in triplicates with ELISA. Data is presented as a mean±SD (n=7). An unpaired t-test was performed with GraphPad Prism. **p ≤ 0.01, ***p ≤ 0.001.

**SUPPLEMENTARY TABLES**

**Table S1. Differentially regulated proteins in OCT- and control gel-treated wounded skin.** OCT-treated and control gel-treated cultured human TS skin biopsies (age range: 38-45 years; n=5) were tested using LC-MS. An unpaired t-test was performed with Perseus, *p < 0.05. Difference is displayed as log_2_ fold change (Log_2_FC < -1 = downregulated; Log_2_FC > 1 = upregulated in OCT-treated samples), *p-*values are ordered from lowest to highest. Protein function was determined using the Uniprot database. Proteins marked in red were further analysed.

**Table S2. Differentially regulated proteins in OCT-treated and untreated wounded skin.** OCT-treated and untreated cultured human TS skin biopsies (age range: 38-45 years; n=5) were tested using LC-MS. An unpaired t-test was performed with Perseus, *p < 0.05. Difference is displayed as log_2_ fold change (Log_2_FC < -1 = downregulated; Log_2_FC > 1 = upregulated in OCT-treated samples), *p*-values are ordered from lowest to highest. Protein function was determined using the Uniprot database. Proteins marked in red were further analysed.

**Table S3. Differentially regulated proteins in control gel-treated and untreated wounded skin.** Control gel-treated and untreated cultured human TS skin biopsies (age range: 38-45 years; n=5) were tested using LC-MS. An unpaired t-test was performed with Perseus, *p < 0.05. Difference is displayed as log_2_ fold change (Log_2_FC < -1 = downregulated; Log_2_FC > 1 = upregulated in OCT-treated samples), *p*-values are ordered from lowest to highest. Protein function was determined using the Uniprot database. Proteins marked in grey are differentially regulated between OCT-treated and untreated samples, proteins marked in turquoise are differentially regulated between OCT-treated and control gel-treated samples.

**Table S4. Differentially regulated proteins in the epidermal compartment of OCT- and control gel-treated skin.** OCT-treated and control gel-treated cultured human TS skin biopsies (age range: 36-44 years; n=3) were tested using LC-MS. An unpaired t-test was performed with Perseus, *p < 0.05. Difference is displayed as log_2_ fold change (Log_2_FC < -1 = downregulated; Log_2_FC > 1 = upregulated in OCT-treated samples), *p*-values are ordered from lowest to highest. Protein function was determined using the Uniprot database. Indicated are only proteins involved in tissue repair and/or immune response processes with a *p*-value < 0.01.

**Table S5. Differentially regulated proteins in the epidermal compartment of OCT-treated and untreated skin.** OCT-treated and untreated cultured human TS skin biopsies (age range: 36-44 years; n=3) were tested using LC-MS. An unpaired t-test was performed with Perseus, *p < 0.05. Difference is displayed as log_2_ fold change (Log_2_FC < -1 = downregulated; Log_2_FC > 1 = upregulated in OCT-treated samples), *p*-values are ordered from lowest to highest. Protein function was determined using the Uniprot database. Indicated are only proteins involved in tissue repair and/or immune response processes with a *p*-value < 0.01.

**Table S6. Differentially regulated proteins in the dermal compartment of OCT-treated and untreated skin.** OCT-treated and untreated cultured human TS skin biopsies (age range: 36-44 years; n=3) were tested using LC-MS. An unpaired t-test was performed with Perseus, *p < 0.05. Difference is displayed as log_2_ fold change (Log_2_FC < -1 = downregulated; Log_2_FC > 1 = upregulated in OCT-treated samples), *p*-values are ordered from lowest to highest. Protein function was determined using the Uniprot database. Indicated are only proteins involved in tissue repair and/or immune response processes with a *p*-value < 0.01.

**Table S7. Differentially regulated proteins in the dermal compartment of OCT- and control gel-treated skin.** OCT-treated and control gel-treated cultured human TS skin biopsies (age range: 36-44 years; n=3) were tested using LC-MS. An unpaired t-test was performed with Perseus, *p < 0.05. Difference is displayed as log_2_ fold change (Log_2_FC < -1 = downregulated; Log_2_FC > 1 = upregulated in OCT-treated samples), *p*-values are ordered from lowest to highest. Protein function was determined using the Uniprot database. Indicated are only proteins involved in tissue repair and/or immune response processes with a *p*-value < 0.01.

**Table S8. Differentially regulated proteins in the epidermal and dermal compartment of control gel-treated and untreated skin.** Control gel-treated and untreated cultured human TS skin biopsies (age range: 36-44 years; n=3) were tested using LC-MS. An unpaired t-test was performed with Perseus, *p < 0.05. Difference is displayed as log_2_ fold change (Log_2_FC < -1 = downregulated; Log_2_FC > 1 = upregulated in OCT-treated samples), *p*-values are ordered from lowest to highest. Protein function was determined using the Uniprot database. Proteins marked in grey are differentially regulated between OCT-treated and untreated epidermal samples, proteins marked in turquoise are differentially regulated between OCT-treated and control gel-treated dermal samples.
