# Supplementary material for: Octenidine-based hydrogel shows anti-inflammatory and protease-inhibitory capacities in wounded human skin
Source: Sci Rep. 2021 Jan 8;11:32. doi: 10.1038/s41598-020-79378-9 (PMC7794247; doi:10.1038/s41598-020-79378-9)
Supplement: Supplementary file 2 — Supplementary figures and tables. [file 41598_2020_79378_MOESM2_ESM.docx]

**Octenidine-based hydrogel shows anti-inflammatory and protease-inhibitory capacities in wounded human skin**

**Saskia Seiser^1*^, Lukas Janker^2,3*^, Nina Zila^1^, Michael Mildner^1^, Ana Rakita^1^, Johannes Matiasek^4^, Andrea Bileck^2,3^, Christopher Gerner^2,3^, Verena Paulitschke^1^, Adelheid Elbe-Bürger^1§^**

1Department of Dermatology, Medical University of Vienna, Vienna, Austria

2Department of Analytical Chemistry, University of Vienna, Vienna, Austria

3Joint Metabolome Facility, University of Vienna and Medical University of Vienna, Vienna, Austria

4Department of Plastic, Aesthetic and Reconstructive Surgery, St. Josef Hospital, Vienna, Austria

*share first authorship

^§^corresponding author; e-mail: adelheid.elbe-buerger@meduniwien.ac.at

**Figure S1**

**
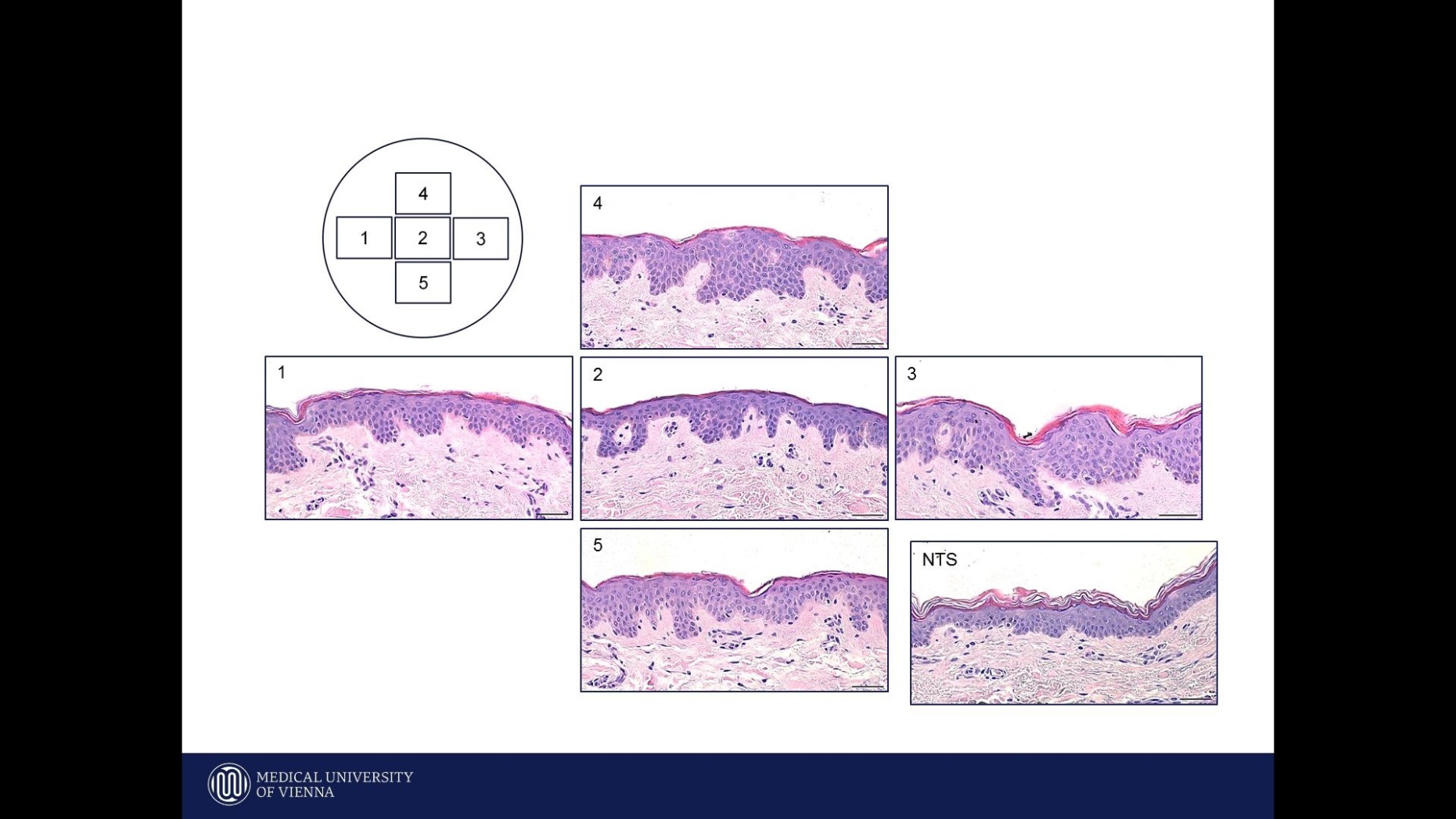
**

**Figure S2**

**
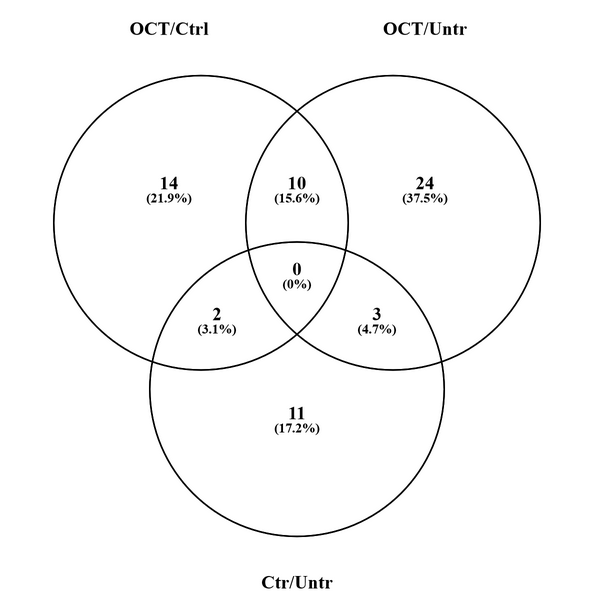
**

**Figure S3**


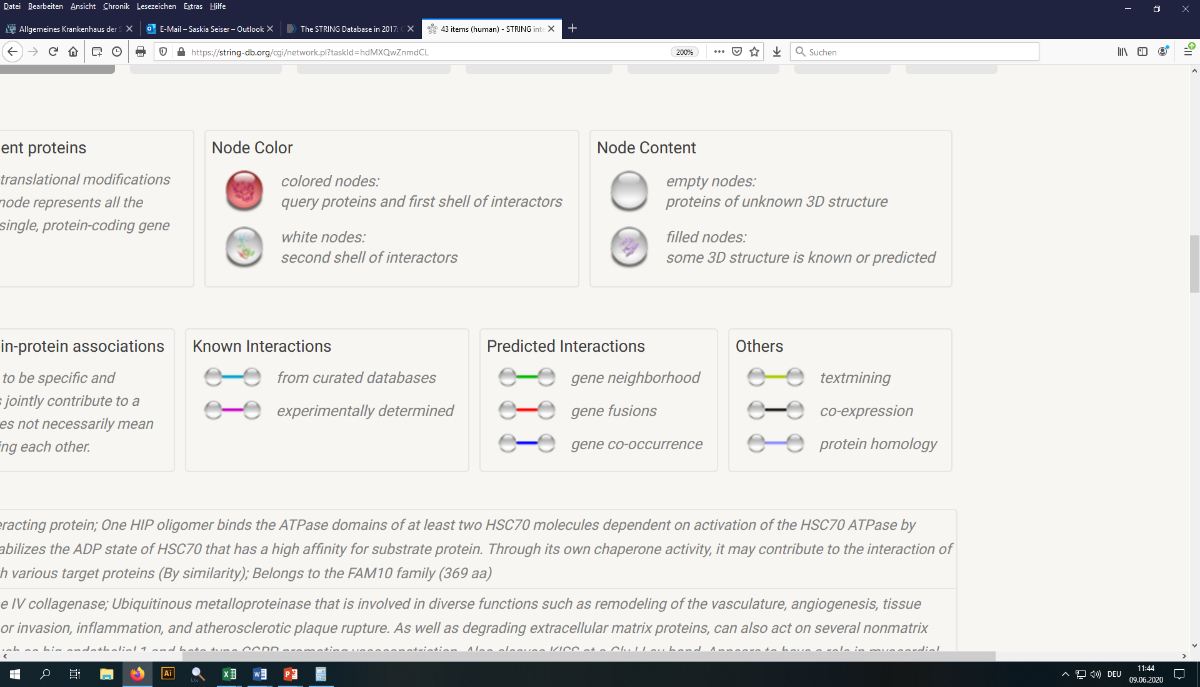

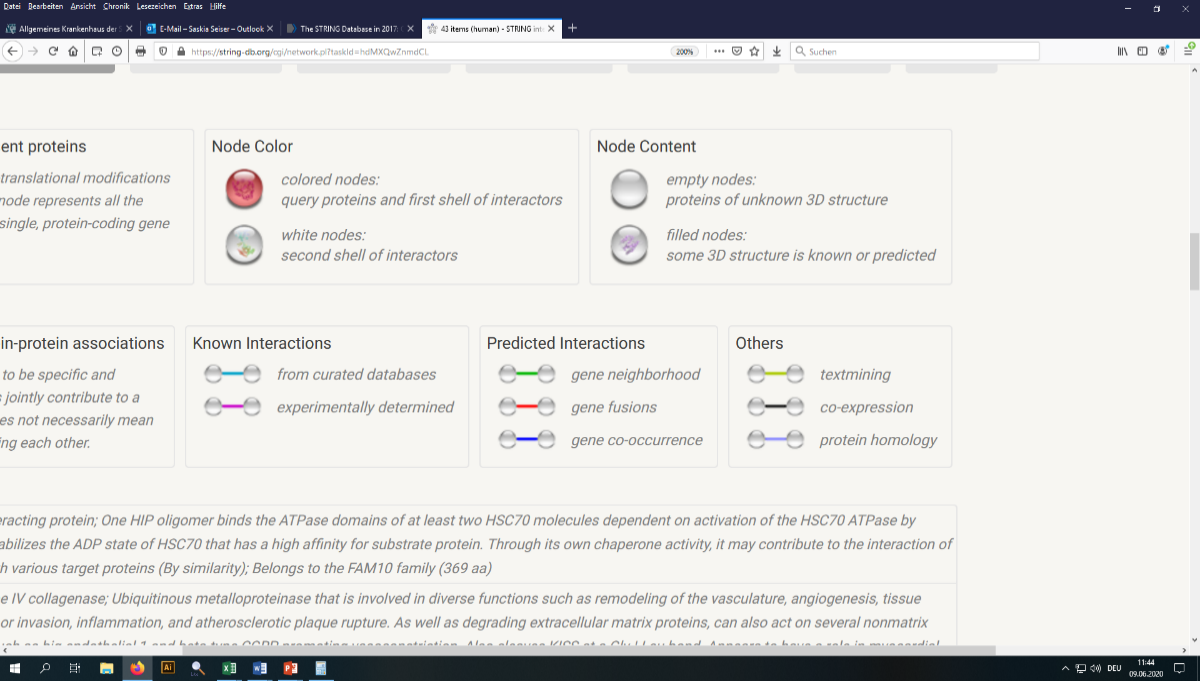

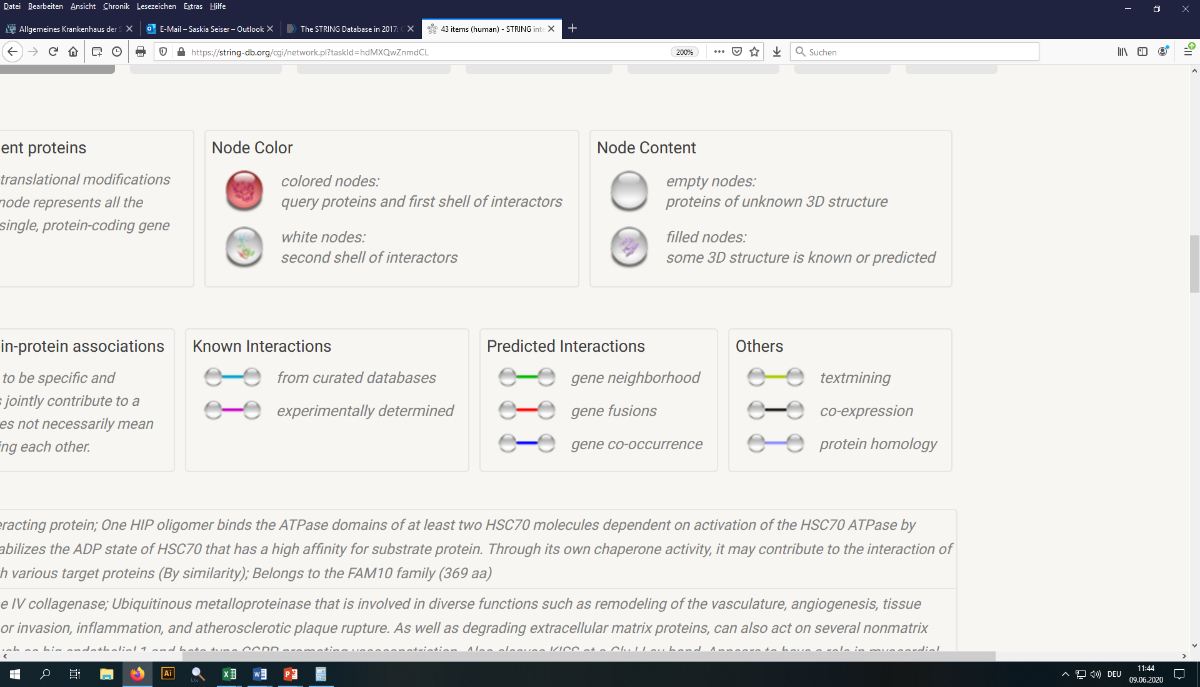

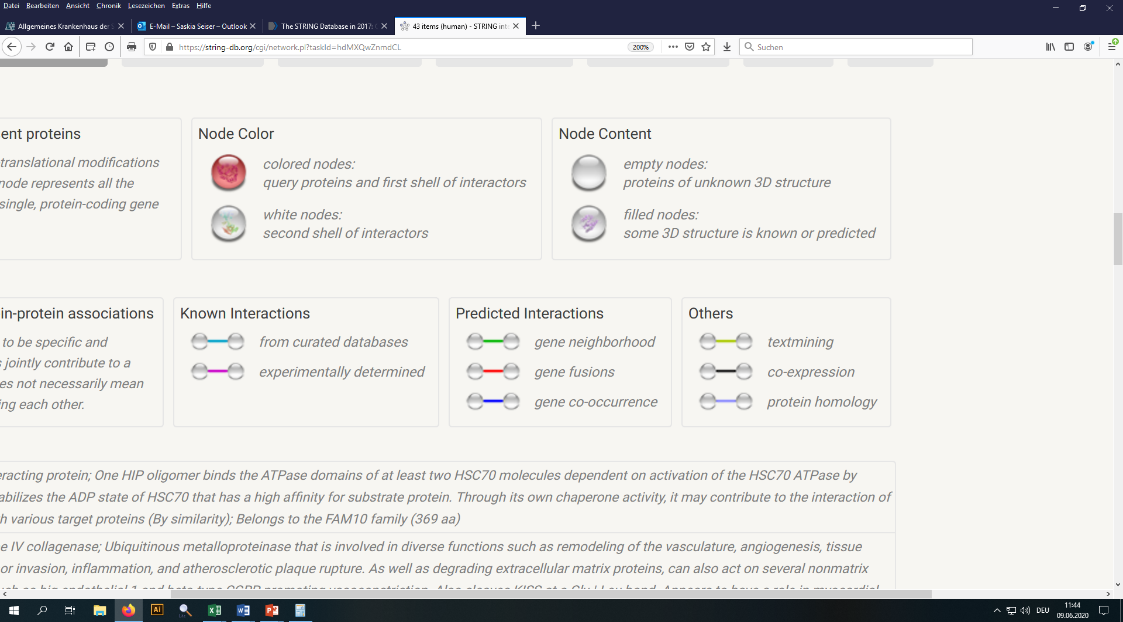

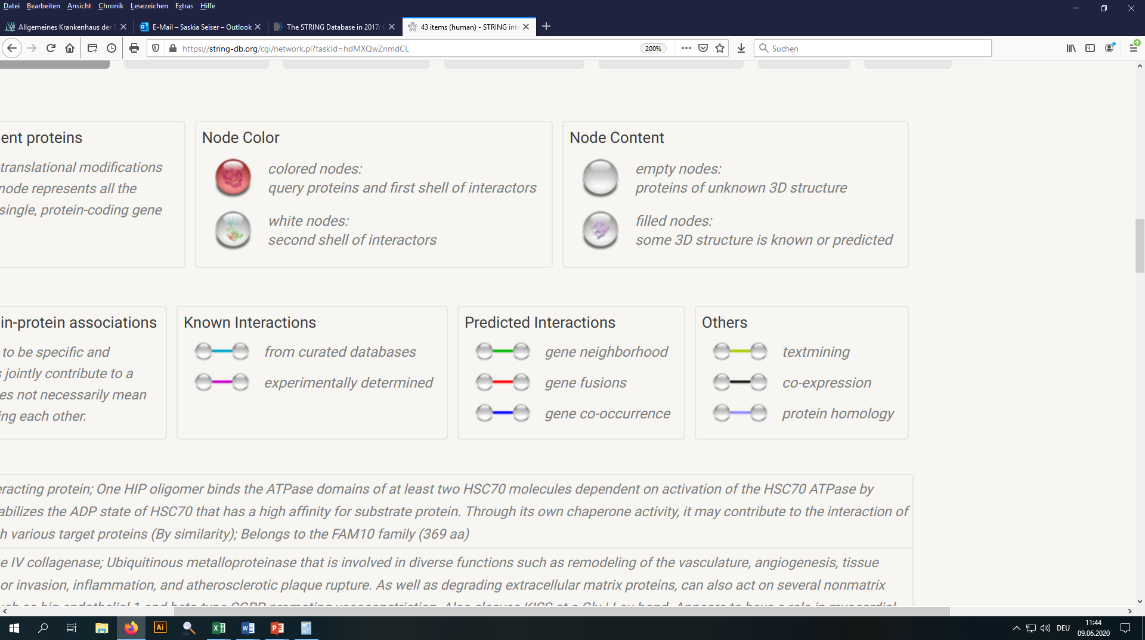

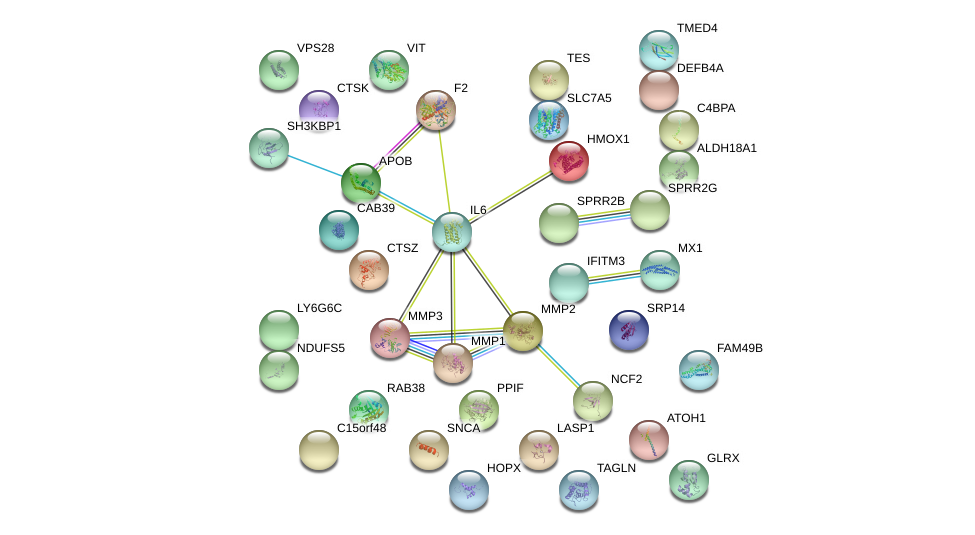


**Figure S4**

**
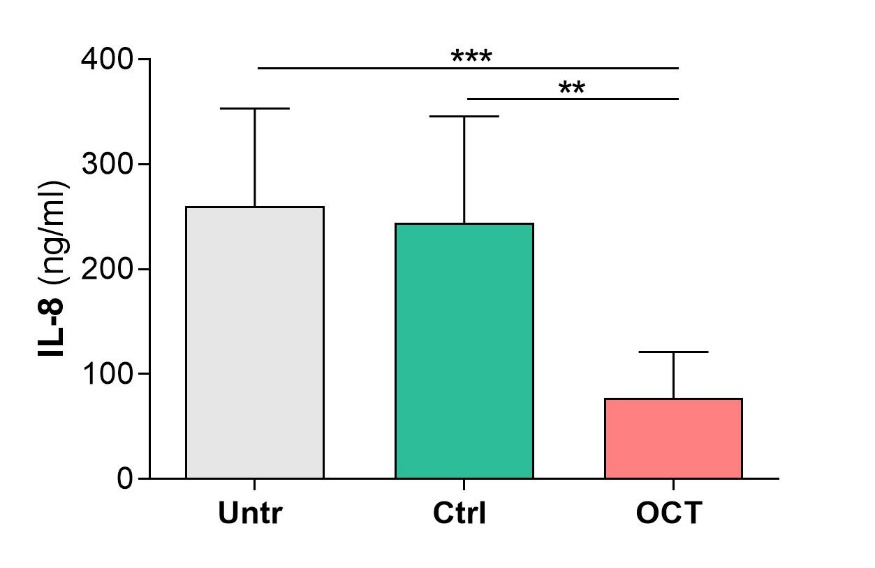
**

**Table S1. Differentially regulated proteins in OCT- and control gel-treated wounded skin**

| **Acc. Nr.** | **Gene names** | **Protein names** | **Difference** | ***p*-value** |
| --- | --- | --- | --- | --- |
| Q15418 | RPS6KA1 | Ribosomal protein S6 kinase alpha-1 | 2.72936 | 0.0406338 |
| P57729 | RAB38 | Ras-related protein Rab-38 | -2.31488 | 0.0024860 |
| Q5T5C0 | STXBP5 | Syntaxin-binding protein 5 | -2.16046 | 0.0441092 |
| Q96NZ8 | WFIKKN1 | WAP, Kazal, immunoglobulin, Kunitz and NTR domain-containing protein 1 | -2.05995 | 0.0460281 |
| Q96B49 | TOMM6 | Mitochondrial import receptor subunit TOM6 homolog | -1.89169 | 0.0123562 |
| P37108 | SRP14 | Signal recognition particle 14 kDa protein | -1.79208 | 0.0164122 |
| P07196 | NEFL | Neurofilament light polypeptide | -1.78873 | 0.0307429 |
| Q9BPY8 | HOPX | Homeodomain-only protein | -1.66531 | 0.0057730 |
| Q8N766 | EMC1 | ER membrane protein complex subunit 1 | 1.58853 | 0.0429446 |
| O14618 | CCS | Copper chaperone for superoxide dismutase | -1.5529 | 0.0442306 |
| P49593 | PPM1F | Protein phosphatase 1F | -1.54092 | 0.0150490 |
| Q9BSD7 | NTPCR | Cancer-related nucleoside-triphosphatase | 1.22018 | 0.0147584 |
| P23610 | F8A1 | Factor VIII intron 22 protein | 1.19871 | 0.0161568 |
| P35442 | THBS2 | Thrombospondin-2 | -1.10232 | 0.0452981 |
| Q8TEX9 | IPO4 | Importin-4 | 1.01008 | 0.0403239 |
| Proteins involved in tissue repair processes | | | | |
| Q96B97 | SH3KBP1 | SH3 domain-containing kinase-binding protein 1 | -2.50139 | 0.0010320 |
| O75190 | DNAJB6 | DnaJ homolog subfamily B member 6 | -2.42276 | 0.0000329 |
| Q9BYE4 | SPRR2G | Small proline-rich protein 2G | -1.29277 | 0.0014479 |
| Q9Y696 | CLIC4 | Chloride intracellular channel protein 4 | -1.09563 | 0.0126506 |
| Proteins involved in immune response | | | | |
| P05231 | IL6 | Interleukin-6 | -2.41779 | 0.0081152 |
| Q99538 | LGMN | Legumain | 2.20007 | 0.0198880 |
| P20591 | MX1 | Interferon-induced GTP-binding protein Mx1 | -1.35853 | 0.0097801 |
| O95867 | LY6G6C | Lymphocyte antigen 6 complex locus protein G6c | -1.3394 | 0.0168512 |
| Q9C002 | NMES1 | Normal mucosa of esophagus-specific gene 1 protein | -1.18638 | 0.0169618 |
| P61026 | RAB10 | Ras-related protein Rab-10 | -1.09958 | 0.0363097 |
| Proteins involved in both, tissue repair and immune response | | | | |
| P03956 | MMP1 | Interstitial collagenase | -1.26249 | 0.0011988 |

**Table S2. Differentially regulated proteins in OCT-treated and untreated wounded skin**

| **Acc. Nr.** | **Gene names** | **Protein names** | **Difference** | ***p*-value** |
| --- | --- | --- | --- | --- |
| Q92858 | ATOH1 | Protein atonal homolog 1 | -3.12793 | 0.0002307 |
| Q7Z7H5 | TMED4 | Transmembrane emp24 domain-containing protein 4 | 2.39738 | 0.0446175 |
| Q9BPY8 | HOPX | Homeodomain-only protein | -2.38045 | 0.0005303 |
| P57729 | RAB38 | Ras-related protein Rab-38 | -2.13996 | 0.0019076 |
| O43920 | NDUFS5 | NADH dehydrogenase [ubiquinone] iron-sulfur protein 5 | -1.98058 | 0.0064109 |
| Q14847 | LASP1 | LIM and SH3 domain protein 1 | -1.90196 | 0.0219411 |
| Q9UBR2 | CTSZ | Cathepsin Z | -1.68655 | 0.0419445 |
| P37108 | SRP14 | Signal recognition particle 14 kDa protein | -1.50146 | 0.0324033 |
| P30405 | PPIF | Peptidyl-prolyl cis-trans isomerase F, mitochondrial | -1.42846 | 0.0401322 |
| P35754 | GLRX | Glutaredoxin-1 | -1.3358 | 0.0012070 |
| P54886 | ALDH18A1 | Delta-1-pyrroline-5-carboxylate synthase | -1.23201 | 0.0453780 |
| Q9Y376 | CAB39 | Calcium-binding protein 39 | -1.18201 | 0.0004578 |
| Q01650 | SLC7A5 | Large neutral amino acids transporter small subunit 1 | -1.15907 | 0.0284621 |
| Q9UK41 | VPS28 | Vacuolar protein sorting-associated protein 28 homolog | -1.15247 | 0.0438657 |
| Q9UGI8 | TES | Testin | -1.09905 | 0.0330494 |
| Proteins involved in tissue repair processes | | | | |
| Q96B97 | SH3KBP1 | SH3 domain-containing kinase-binding protein 1 | -2.99325 | 0.0023696 |
| P43235 | CTSK | Cathepsin K | -2.27851 | 0.0065599 |
| P35325 | SPRR2B | Small proline-rich protein 2B | -1.60542 | 0.0435721 |
| Q9BYE4 | SPRR2G | Small proline-rich protein 2G | -1.41857 | 0.0008890 |
| Q01995 | TAGLN | Transgelin | -1.06854 | 0.0483124 |
| Q6UXI7 | VIT | Vitrin | 1.05801 | 0.0498010 |
| proteins involved in immune response | | | | |
| P05231 | IL6 | Interleukin-6 | -2.12485 | 0.0259179 |
| Q01628 | IFITM3 | Interferon-induced transmembrane protein 3 | -1.90649 | 0.0366628 |
| Q9C002 | NMES1 | Normal mucosa of esophagus-specific gene 1 protein | -1.49802 | 0.0091243 |
| P20591 | MX1 | Interferon-induced GTP-binding protein Mx1 | -1.35044 | 0.0033264 |
| O15263 | DEFB4A | Beta-defensin 4A | -1.16194 | 0.0110583 |
| Q9NUQ9 | FAM49B | Protein FAM49B | -1.14202 | 0.0358444 |
| P04003 | C4BPA | C4b-binding protein alpha chain | 1.11854 | 0.0386461 |
| O95867 | LY6G6C | Lymphocyte antigen 6 complex locus protein G6c | 1.94051 | 0.0123234 |
| proteins involved in both, tissue repair and immune response | | | | |
| P03956 | MMP1 | Interstitial collagenase | -1.54141 | 0.0002663 |
| P09601 | HMOX1 | Heme oxygenase 1 | -1.68656 | 0.0313425 |
| P37840 | SNCA | Alpha-synuclein | -1.43251 | 0.0167263 |
| P08254 | MMP3 | Stromelysin-1 | -1.42857 | 0.0184192 |
| P19878 | NCF2 | Neutrophil cytosol factor 2 | -1.32954 | 0.0228254 |
| P08253 | MMP2 | 72 kDa type IV collagenase | -1.02733 | 0.0072739 |
| P00734 | F2 | Prothrombin | 1.26839 | 0.0023982 |
| P04114 | APOB | Apolipoprotein B-100 | 1.71104 | 0.0305505 |

**Table S3. Differentially regulated proteins in control gel-treated and untreated wounded skin**

| **Acc. Nr.** | **Gene names** | **Protein names** | **Difference** | ***p*-value** |
| --- | --- | --- | --- | --- |
| Q92858 | ATOH1 | Protein atonal homolog 1 | 2.90260 | 0.0467329 |
| Q8N4B1 | FAM109A | Sesquipedalian-1 | 1.99453 | 0.0338739 |
| Q15418 | RPS6KA1 | Ribosomal protein S6 kinase alpha-1 | 1.31693 | 0.0181723 |
| P57735 | RAB25 | Ras-related protein Rab-25 | 1.11329 | 0.0214313 |
| Q5BKU9 | OXLD1 | Oxidoreductase-like domain-containing protein 1 | 1.05709 | 0.0120118 |
| P51911 | CNN1 | Calponin-1 | -1.01570 | 0.0274797 |
| Q14839 | CHD4 | Chromodomain-helicase-DNA-binding protein 4 | -1.07617 | 0.0420545 |
| Q9UBX7 | KLK11 | Kallikrein-11 | -1.45470 | 0.0382948 |
| Q8IW45 | CARKD | ATP-dependent (S)-NAD(P)H-hydrate dehydratase | -1.92203 | 0.0332909 |
| Q2M389 | KIAA1033 | WASH complex subunit 7 | 1.11104 | 0.0472920 |
| O95445 | APOM | Apolipoprotein M | -2.89068 | 0.0386007 |
| Q8TEX9 | IPO4 | Importin-4 | -2.99654 | 0.0314189 |
| **Proteins involved in tissue repair processes** | | | | |
| P21941 | MATN1 | Cartilage matrix protein | 3.74950 | 0.0031799 |
| Q6UXI7 | VIT | Vitrin | 1.10172 | 0.0103093 |
| **Proteins involved in immune response** | | | | |
| Q01650 | SLC7A5 | Large neutral amino acids transporter small subunit 1 | -1.12076 | 0.0198887 |
| Q9BT09 | CNPY3 | Protein canopy homolog 3 | -1.74096 | 0.0000938 |

**Table S4. Differentially regulated proteins in the epidermal compartment of OCT- and control gel-treated skin**

| **Acc. Nr.** | **Gene names** | **Protein names** | **Difference** | ***p*-value** |
| --- | --- | --- | --- | --- |
| **Proteins involved in tissue repair processes** | | | | |
| Q13480 | GAB1 | GRB2-associated-binding protein 1 | 2.65622 | 0.0000351 |
| P40126 | TYRP2 | L-dopachrome tautomerase | -1.75076 | 0.0080068 |
| P30876 | RPB2 | DNA-directed RNA polymerase II subunit RPB2 | -1.07747 | 0.0030789 |
| **Proteins involved in immune response** | | | | |
| O14576 | DC1I1 | Cytoplasmic dynein 1 intermediate chain 1 | -2.13067 | 0.0077364 |
| P50238 | CRIP1 | Cysteine-rich protein 1 | -1.60604 | 0.0091073 |
| Q9Y6K5 | OAS3 | 2'-5'-oligoadenylate synthase 3 | 1.55990 | 0.0078750 |
| P26583 | HMGB2 | High mobility group protein B2 | -1.39601 | 0.0003445 |
| Q9H9Q4 | NHEJ1 | Non-homologous end-joining factor 1 | -1.39500 | 0.0062450 |
| O94855 | SC24D | Protein transport protein Sec24D | 1.39158 | 0.0088761 |
| **Proteins involved in tissue repair and immune response** | | | | |
| Q04206 | TF65 | Transcription factor p65 | 1.29788 | 0.0003202 |

**Table S5. Differentially regulated proteins in the epidermal compartment of OCT-treated and untreated skin**

| **Acc. Nr.** | **Gene names** | **Protein names** | **Difference** | ***p*-value** |
| --- | --- | --- | --- | --- |
| **Proteins involved in tissue repair processes** | | | | |
| Q13480 | GAB1 | GRB2-associated-binding protein 1 | 2.00244 | 0.0093988 |
| **Proteins involved in immune response** | | | | |
| P50238 | CRIP1 | Cysteine-rich protein 1 | -1.67531 | 0.0079399 |
| P42898 | MTHR | Methylenetetrahydrofolate reductase | 2.97058 | 0.0069516 |
| O94855 | SC24D | Protein transport protein Sec24D | 1.96976 | 0.0065865 |
| P26583 | HMGB2 | High mobility group protein B2 | -1.10077 | 0.0065148 |
| P05161 | ISG15 | Ubiquitin-like protein ISG15 | -2.51395 | 0.0055573 |
| O95867 | LY66C | Lymphocyte antigen 6 complex locus protein G6c | 1.78426 | 0.0036757 |
| P05067 | A4 | Amyloid-beta precursor protein | -1.50652 | 0.0004426 |

**Table S6. Differentially regulated proteins in the dermal compartment of OCT-treated and untreated skin**

| **Acc. Nr.** | **Gene names** | **Protein names** | **Difference** | ***p*-value** |
| --- | --- | --- | --- | --- |
| **Proteins involved in tissue repair processes** | | | | |
| P56192 | SYMC | Methionine--tRNA ligase, cytoplasmic | -2.94322 | 0.006886 |
| Q01995 | TAGL | Transgelin | -2.90751 | 0.000173 |
| Q9UBR2 | CATZ | Cathepsin Z | -2.53883 | 0.000414 |
| P21291 | CSRP1 | Cysteine and glycine-rich protein 1 | -2.23585 | 0.000004 |
| Q9NVD7 | PARVA | Alpha-parvin | -2.04670 | 0.000009 |
| O94760 | DDAH1 | N(G),N(G)-dimethylarginine dimethylaminohydrolase 1 | -2.01723 | 0.007180 |
| P13861 | KAP2 | cAMP-dependent protein kinase type II-alpha regulatory subunit | -1.67402 | 0.004539 |
| P19971 | TYPH | Thymidine phosphorylase | -1.58886 | 0.002436 |
| Q96AC1 | FERM2 | Fermitin family homolog 2 | -1.55430 | 0.003212 |
| P12955 | PEPD | Xaa-Pro dipeptidase | -1.51804 | 0.000000 |
| P23381 | SYWC | Tryptophan--tRNA ligase, cytoplasmic | -1.51312 | 0.000935 |
| Q9BUT1 | BDH2 | 3-hydroxybutyrate dehydrogenase type 2 | -1.37434 | 0.002510 |
| Q07157 | ZO1 | Tight junction protein ZO-1 | -1.26865 | 0.003675 |
| P17174 | AATC | Aspartate aminotransferase, cytoplasmic | -1.21726 | 0.002259 |
| P16152 | CBR1 | Carbonyl reductase [NADPH] 1 | -1.16584 | 0.006820 |
| O14773 | TPP1 | Tripeptidyl-peptidase 1 | -1.14612 | 0.008816 |
| P21333 | FLNA | Filamin-A | -1.12147 | 0.000025 |
| P55786 | PSA | Puromycin-sensitive aminopeptidase | -1.08291 | 0.003088 |
| P00558 | PGK1 | Phosphoglycerate kinase 1 | -1.07120 | 0.006314 |
| P07737 | PROF1 | Profilin-1 | -1.06118 | 0.002302 |
| O00299 | CLIC1 | Chloride intracellular channel protein 1 | -1.00769 | 0.001064 |
| **Proteins involved in immune response** | | | | |
| O14684 | PTGES | Prostaglandin E synthase | -2.63726 | 0.0034558 |
| Q92841 | DDX17 | Probable ATP-dependent RNA helicase DDX17 | -2.34598 | 0.0008377 |
| O94855 | SC24D | Protein transport protein Sec24D | -2.22605 | 0.0055047 |
| Q92598 | HS105 | Heat shock protein 105 kDa | -2.17689 | 0.0039631 |
| O00584 | RNT2 | Ribonuclease T2 | -2.08322 | 0.0056919 |
| P50502 | F10A1 | Hsc70-interacting protein | -1.98708 | 0.0052170 |
| Q9BZZ2 | SN | Sialoadhesin | 1.97282 | 0.0095737 |
| Q13451 | FKBP5 | Peptidyl-prolyl cis-trans isomerase FKBP5 | -1.96579 | 0.0068919 |
| Q14019 | COTL1 | Coactosin-like protein | -1.84080 | 0.0074469 |
| Q12846 | STX4 | Syntaxin-4 | -1.74888 | 0.0016726 |
| Q86U42 | PABP2 | Polyadenylate-binding protein 2 | -1.67606 | 0.0024224 |
| P09211 | GSTP1 | Glutathione S-transferase P | -1.51409 | 0.0006849 |
| P61077 | UB2D3 | Ubiquitin-conjugating enzyme E2 D3 | -1.44377 | 0.0006054 |
| Q15436 | SC23A | Protein transport protein Sec23A | -1.40439 | 0.0017876 |
| O95747 | OXSR1 | Serine/threonine-protein kinase OSR1 | 1.38121 | 0.0063146 |
| P04406 | G3P | Glyceraldehyde-3-phosphate dehydrogenase | -1.34162 | 0.0008971 |
| Q9UL46 | PSME2 | Proteasome activator complex subunit 2 | -1.33391 | 0.0028324 |
| P0CG47 | UBB | Polyubiquitin-B | -1.28325 | 0.0063860 |
| P62937 | PPIA | Peptidyl-prolyl cis-trans isomerase A | -1.23512 | 0.0016956 |
| P09661 | RU2A | U2 small nuclear ribonucleoprotein A' | -1.22781 | 0.0000885 |
| P30520 | PURA2 | Adenylosuccinate synthetase isozyme 2 | -1.22047 | 0.0007854 |
| P11142 | HSP7C | Heat shock cognate 71 kDa protein | -1.10203 | 0.0021443 |
| P11766 | ADHX | Alcohol dehydrogenase class-3 | -1.09246 | 0.0072677 |
| O94979 | SC31A | Protein transport protein Sec31A | -1.08827 | 0.0090472 |
| P13693 | TCTP | Translationally-controlled tumor protein | -1.07958 | 0.0038294 |
| P00441 | SODC | Superoxide dismutase [Cu-Zn] | -1.06168 | 0.0051658 |
| **Proteins involved in tissue repair and immune response** | | | | |
| P55008 | AIF1 | Allograft inflammatory factor 1 | -2.00399 | 0.0030479 |
| P07858 | CATB | Cathepsin B | -2.00399 | 0.0039826 |
| P03956 | MMP1 | Interstitial collagenase | -1.88942 | 0.0033735 |
| P51452 | DUS3 | Dual specificity protein phosphatase 3 | -1.85337 | 0.0052820 |
| Q9Y624 | JAM1 | Junctional adhesion molecule A | 1.65474 | 0.0016910 |
| P14780 | MMP9 | Matrix metalloproteinase-9 | -1.63819 | 0.0011585 |
| Q86UX7 | URP2 | Fermitin family homolog 3 | -1.59003 | 0.0003360 |
| P19174 | PLCG1 | 1-phosphatidylinositol 4,5-bisphosphate phosphodiesterase gamma-1 | -1.57649 | 0.0028109 |
| P48357 | LEPR | Leptin receptor | 1.55480 | 0.0088802 |
| P42224 | STAT1 | Signal transducer and activator of transcription 1-alpha/beta | -1.52244 | 0.0023136 |
| P08253 | MMP2 | 72 kDa type IV collagenase | -1.40932 | 0.0075503 |
| P04075 | ALDOA | Fructose-bisphosphate aldolase A | -1.22280 | 0.0021401 |
| P18206 | VINC | Vinculin | -1.18235 | 0.0000531 |
| P07711 | CATL1 | Cathepsin L1 | -1.10075 | 0.0096080 |
| P26006 | ITA3 | Integrin alpha-3 | 1.03767 | 0.0029981 |

**Table S7. Differentially regulated proteins in the dermal compartment of OCT- and control gel-treated skin**

| **Acc. Nr.** | **Gene names** | **Protein names** | **Difference** | ***p*-value** |
| --- | --- | --- | --- | --- |
| **Proteins involved in tissue repair processes** | | | | |
| Q13491 | GPM6B | Neuronal membrane glycoprotein M6-b | 2.47664 | 0.0026752 |
| P21291 | CSRP1 | Cysteine and glycine-rich protein 1 | -2.38177 | 0.0000986 |
| Q9UBR2 | CATZ | Cathepsin Z | -2.01755 | 0.0022841 |
| Q6UXI7 | VITRN | Vitrin | 1.31395 | 0.0048861 |
| P19971 | TYPH | Thymidine phosphorylase | -1.05160 | 0.0009497 |
| **Proteins involved in tissue repair and immune response** | | | | |
| Q86UX7 | URP2 | Fermitin family homolog 3 | -1.02973 | 0.0046244 |

**Table S8. Differentially regulated proteins in the epidermal and dermal compartment of control gel-treated and untreated skin**

| **Epidermis** | | | | |
| --- | --- | --- | --- | --- |
| **Acc. Nr.** | **Gene names** | **Protein names** | **Difference** | ***p*-value** |
| Q13573 | SNW1 | SNW domain-containing protein 1 | 1.06982 | 0.0058638 |
| Q8NFH5 | NUP35 | Nucleoporin NUP35 | 1.11644 | 0.0088378 |
| **Dermis** | | | | |
| **Acc. Nr.** | **Gene names** | **Protein names** | **Difference** | ***p*-value** |
| O95070 | YIF1A | Protein YIF1A | -1.88296 | 0.0068725 |
| Q03154 | ACY1 | Aminoacylase-1 | -1.86772 | 0.0078767 |
| **Proteins involved in tissue repair processes** | | | | |
| P56192 | SYMC | Methionine--tRNA ligase, cytoplasmic | -2.96737 | 0.0069484 |
| **Proteins involved in immune response** | | | | |
| P09661 | RU2A | U2 small nuclear ribonucleoprotein A' | -1.23095 | 0.0089302 |
| Q14019 | COTL1 | Coactosin-like protein | -1.13469 | 0.0001337 |
| **Proteins involved in tissue repair and immune response** | | | | |
| P19174 | PLCG1 | 1-phosphatidylinositol 4,5-bisphosphate phosphodiesterase gamma-1 | -1.78229 | 0.0035769 |
